# Clinical translation of CRISPR-Cas9 therapeutics in cancer and inherited genetic disorders

**DOI:** 10.3389/fgeed.2026.1868779

**Published:** 2026-09-10

**Authors:** Khushi Bashir, Prathiksha Vasudev, Vikas Chhetri, Devika Prasad Dubhashi, Yash Gurunath Teli, Rhitam Biswas, Sudha Ramaiah, Anand Anbarasu

**Affiliations:** 1 Department of Bio-Medical Sciences, School of Bio-Sciences and Technology (SBST), Vellore Institute of Technology (VIT), Vellore, Tamil Nadu, India; 2 Department of Biotechnology, SBST, VIT, Vellore, Tamil Nadu, India; 3 Department of Bio-Sciences, SBST, VIT, Vellore, Tamil Nadu, India

**Keywords:** cancer, clinical translation, CRISPR-Cas9, gene therapy, genome editing, inherited genetic disorders

## Abstract

CRISPR-Cas9, adapted from the bacterial Type II CRISPR adaptive immune system, functions as a programmable RNA-guided endonuclease that employs a single-guide RNA to direct Cas9 to specific genomic loci. CRISPR-Cas9 has transformed targeted genome editing by replacing complex protein engineering with programmable Watson–Crick base pairing between the guide RNA and target DNA. This review was developed following a structured literature search of major biomedical databases and clinical trial registries to synthesize current evidence on the therapeutic applications of CRISPR-Cas9 in oncology and inherited genetic disorders. Clinical studies of *ex vivo BCL11A*-enhancer editing have shown fetal hemoglobin reactivation, with most evaluable participants with sickle cell disease remaining free of severe vaso-occlusive crises for the prespecified period and most evaluable participants with transfusion-dependent β-thalassemia achieving sustained transfusion independence. *In vivo* reductions in circulating transthyretin protein levels have been achieved for transthyretin amyloidosis via lipid nanoparticle delivery, while clinically meaningful improvements in selected measures of visual function were observed in a subset of patients receiving subretinal AAV-delivered CRISPR editing for *CEP290*-associated Leber congenital amaurosis type 10. Preclinical and early clinical studies have further investigated CRISPR-engineered T cells designed to improve antitumor activity, persistence, or resistance to inhibitory signaling. Despite these advances, key translational hurdles include the risk of off-target mutations and large-scale chromosomal rearrangements. Furthermore, immune responses against bacterial Cas9 nucleases and viral delivery vectors may limit the long-term efficacy of CRISPR-based therapies, while technical barriers surrounding delivery to extrahepatic tissues, such as skeletal muscle and the central nervous system, continue to hinder broader clinical success. Ethical concerns regarding germline modifications and the high cost of individualized therapies present additional translational challenges. Consequently, emerging DSB-independent technologies, such as base editing and prime editing, may reduce selected DSB-associated liabilities, but each introduces distinct editing, delivery, and genotoxicity risks that require product-specific evaluation.

## 1 Introduction

### 1.1 Historical overview of genome editing

The development of genome editing began with protein-based designer nucleases, including meganucleases, zinc finger nucleases (ZFNs), and transcription activator-like effector nucleases (TALENs). TALENs utilize TALE repeats that provide a simple and predictable one-to-one nucleotide recognition code for single base pairs, whereas ZFNs rely on complex modular architectures in which individual zinc finger domains recognize specific 3-bp DNA triplets (Gaj et al., 2013). Although these earlier platforms required extensive target-specific protein engineering, they established the feasibility of programmable genome modification and provided an important technological foundation for subsequent therapeutic genome editing strategies. However, their broader adoption was constrained by the need for target-specific protein engineering, context-dependent activity, and the potential for off-target cleavage and associated cytotoxicity (Gaj et al., 2013).

**FIGURE 1:**
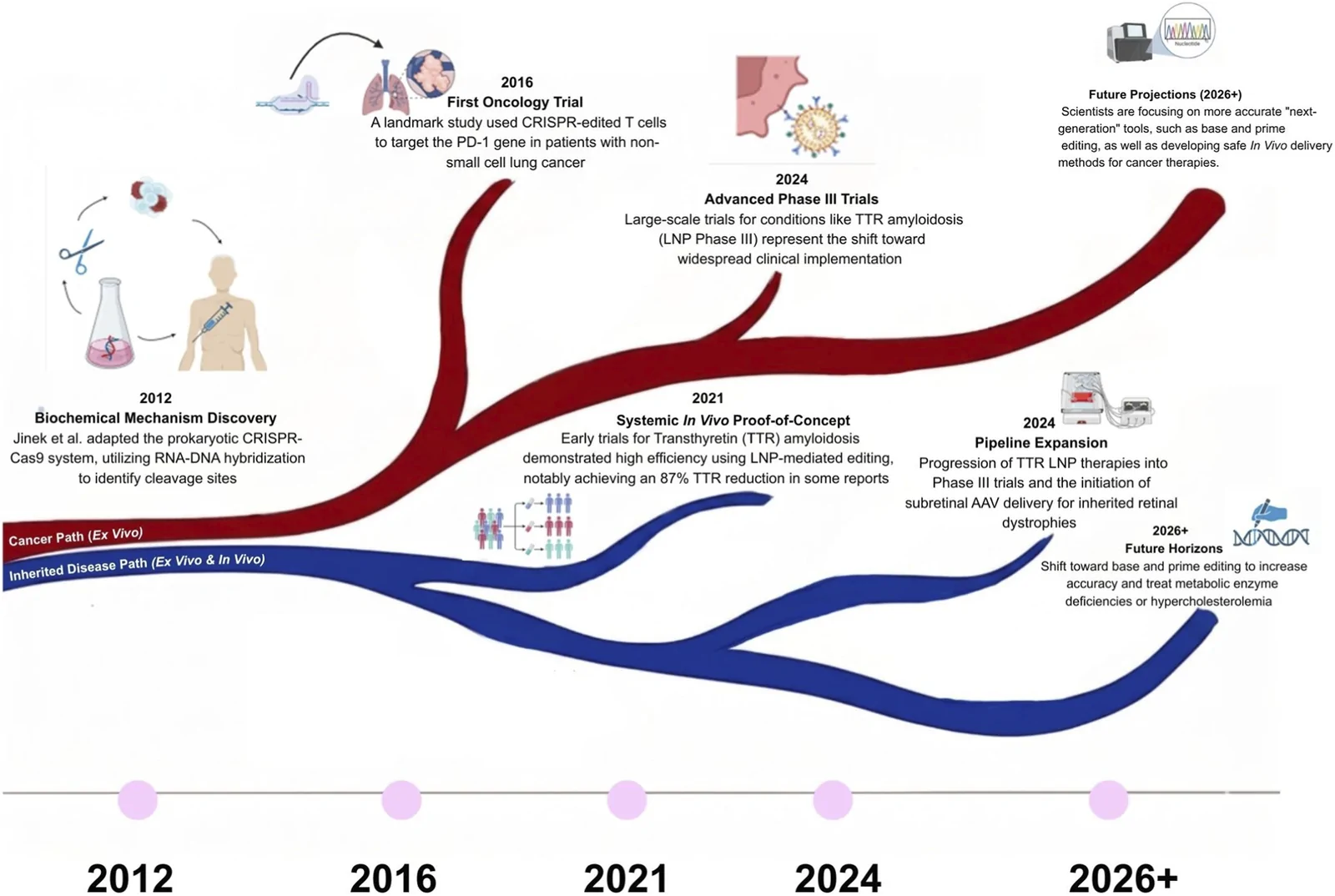
Timeline of CRISPR therapeutics milestones. Key milestones in CRISPR–Cas9 therapeutic development (2012–2026) across cancer (red) and inherited diseases (blue), including early mechanistic discovery, first-in-human trials, *in vivo* delivery advances, and regulatory approval of CRISPR-edited therapies for hemoglobinopathies, and late-stage clinical development of *in vivo TTR* editing for ATTR amyloidosis. The timeline highlights rapid clinical translation and emerging next-generation editing strategies.

The successful adaptation of the prokaryotic CRISPR-Cas9 system to eukaryotic cells between 2012 and 2013 represented a paradigm shift in genome editing. Unlike its predecessors, CRISPR-Cas9 separated the targeting mechanism from the nuclease itself by employing RNA-DNA Watson–Crick base pairing between the guide RNA (gRNA) spacer and the target protospacer, rather than relying on complex protein-DNA interactions (Mengstie and Wondimu, 2021). This modular architecture substantially simplified genome editing by enabling retargeting through modification of a short 20-nucleotide sequence within the single-guide RNA (sgRNA) rather than protein engineering. This innovation facilitated multiplex genome editing and high-throughput functional genomic screening that were technically prohibitive using earlier nuclease platforms (Adli, 2018). Moreover, these tools enabled the rapid generation of heritable mutations in numerous model organisms, ranging from zebrafish to non-human primates, with efficiencies previously difficult to achieve using conventional homologous recombination. Consequently, CRISPR-Cas9 rapidly became the predominant genome editing platform for both basic research and therapeutic development. Figure 1 summarizes key milestones in the clinical translation of CRISPR therapeutics.

### 1.2 CRISPR-Cas9 mechanism and platforms

The Class 2 Type II CRISPR-Cas9 system, most commonly represented by *Streptococcus pyogenes* Cas9 (SpCas9), functions as a programmable ribonucleoprotein (RNP) complex. The Cas9 protein is structurally divided into a recognition (REC) lobe, which binds sgRNA, and a nuclease (NUC) lobe, which contains the RuvC and HNH domains (Xu and Li, 2020). The system uses the combined action of the Cas9 protein and an sgRNA to direct the enzyme to a target sequence located immediately upstream of a 5′-NGG-3′ protospacer adjacent motif (PAM). Once the PAM is recognized, the Cas9 nuclease causes localized DNA melting and forms an R-loop, positioning its nuclease domains to cleave both DNA strands approximately three nucleotides upstream of the PAM, typically generating blunt or near-blunt double-strand break (DSB) ends (Ding et al., 2023). Cellular DNA repair pathways determine how CRISPR-induced DSBs are resolved: the error-prone non-homologous end joining (NHEJ) pathway frequently introduces small insertions or deletions (indels) that disrupt gene function, whereas homology-directed repair (HDR) utilizes a donor template to enable precise sequence restoration or insertion (Yang et al., 2020).

**FIGURE 2:**
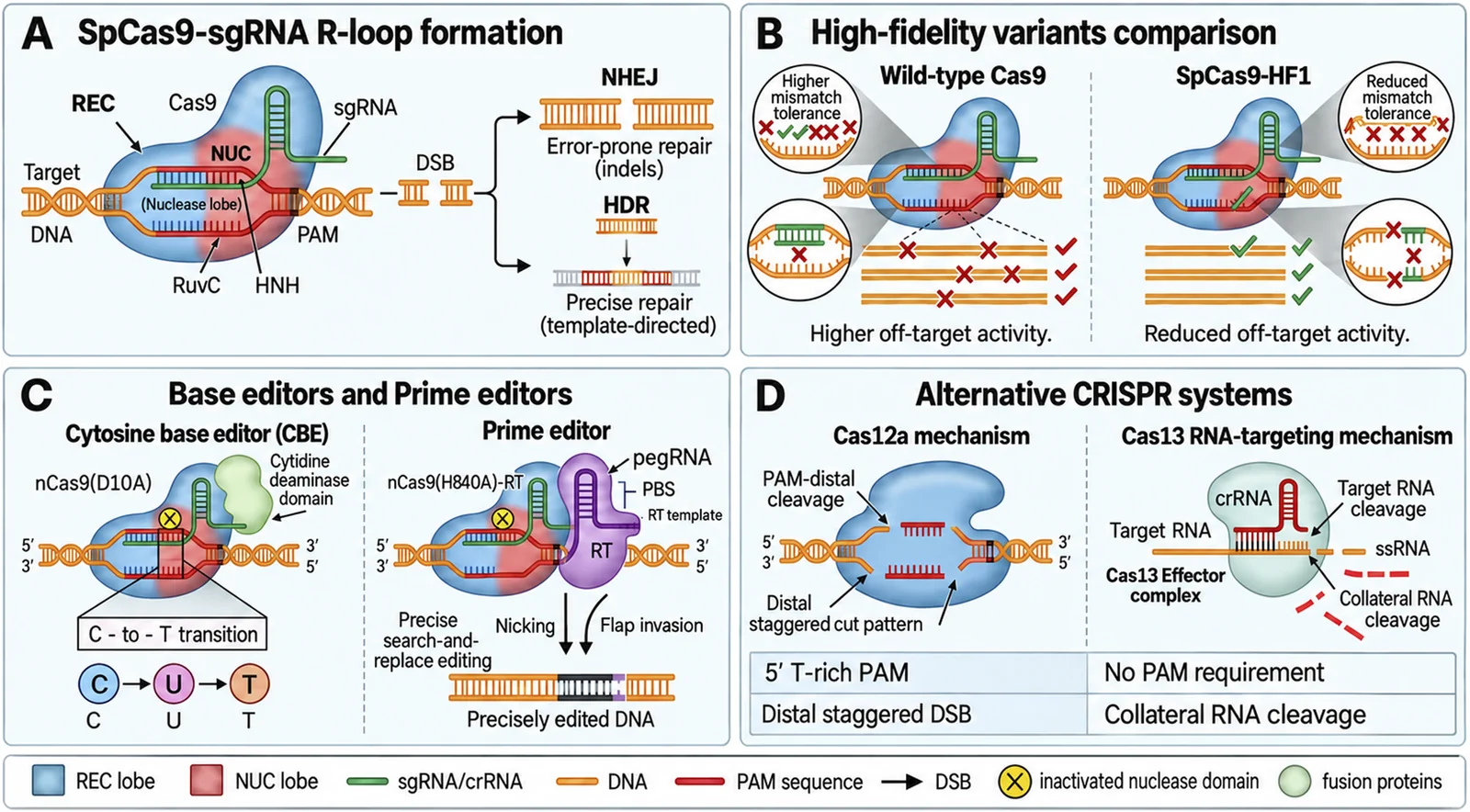
Schematic of CRISPR-Cas9 mechanism and next-gen variants. **(A)** Canonical Cas9-sgRNA targeting, PAM recognition, and double-strand break formation followed by NHEJ or HDR repair. **(B)** High-fidelity Cas9 variants with reduced off-target activity. **(C)** Precision editors, including base and prime editing, enable nucleotide-level modifications without double-strand breaks. **(D)** Alternative systems such as Cas12 and Cas13 expand targeting scope and functionality.

To improve genome editing specificity, high-fidelity Cas9 variants, such as SpCas9-HF1, were engineered via rational design to reduce off-target activity by limiting non-specific DNA contacts (Kleinstiver et al., 2016). Beyond nuclease-mediated DNA cleavage, next-generation CRISPR platforms use catalytically impaired Cas9 variants as programmable scaffolds for precise genome and epigenome engineering. Base editors combine a Cas9 nickase with a deaminase to create point mutations through direct base conversions (such as C-to-T or A-to-G) without inducing DSBs, thereby avoiding the obligate DSBs associated with conventional Cas9 nuclease editing and reducing selected DSB-associated genotoxicity risks (Eid et al., 2018). By incorporating a reverse transcriptase, prime editing enables a broader spectrum of targeted genomic modifications, including insertions and targeted deletions (Zhao et al., 2023). Furthermore, catalytically dead Cas9 (dCas9) can be fused to chromatin-modifying enzymes or transcriptional modulators to regulate gene expression without altering the underlying DNA sequence (La Russa and Qi, 2015). The mechanism of CRISPR-Cas9, along with its next-generation variants, is shown in Figure 2.

### 1.3 Rationale for therapeutic targeting in cancer and inherited diseases

Genome editing is particularly attractive for diseases with well-defined genetic drivers because it directly targets the underlying molecular defect at the genomic level, rather than merely managing downstream phenotypic manifestations (Doudna, 2020). Oncogenesis is driven by the progressive accumulation of genetic alterations, resulting in the constitutive activation of oncogenes (e.g., *KRAS*, *MYC*) and the concurrent inactivation of tumor suppressor genes (e.g., *TP53*, *PTEN*). Together, these changes promote unconstrained proliferation and evasion of apoptosis (Fatemian and Chowdhury, 2014). Similarly, monogenic disorders arise predominantly from pathogenic variants affecting a single gene, making them particularly suitable candidates for targeted genome editing strategies (Maeder and Gersbach, 2016). Traditional pharmacological therapies primarily modulate downstream pathways, whereas RNA interference (RNAi) transiently suppresses target transcripts; by contrast, genome editing can introduce durable modifications at disease-associated genomic loci (Morris and Chan, 2015).

Genome editing offers several distinct advantages over conventional pharmacological therapies and RNAi approaches. Unlike RNAi, which transiently suppresses gene expression at the mRNA level, therapeutic genome editing directly modifies genomic DNA within targeted somatic cells and therefore has the potential to produce durable molecular correction (Li et al., 2025). Notably, these modifications are not transmitted to offspring, as current clinical CRISPR applications strictly involve somatic rather than germline editing (Ryu et al., 2023). It is important to emphasize that durable molecular correction does not necessarily equate to a complete clinical cure; long-term therapeutic benefit relies heavily upon the sustained persistence of edited cells, disease-specific biology, and a durable safety profile (Qie et al., 2025). Additionally, genome editing enables the simultaneous modification of multiple genomic loci through multiplex editing strategies, an approach particularly advantageous for genetically heterogeneous tumors that remain difficult to achieve using RNAi alone. RNAi acts on RNA transcripts, whereas CRISPR nucleases or dCas9-based regulators can be directed to DNA regulatory elements such as promoters and enhancers (Barrangou et al., 2015).

## 2 Methods

### 2.1 Literature search strategy

A structured literature search was conducted to identify studies describing the therapeutic applications of CRISPR-Cas9 genome editing in oncology and inherited genetic disorders. Systematic literature searches were performed using PubMed, Scopus, Web of Science, and Google Scholar, supplemented by searches of ClinicalTrials.gov, to identify completed, ongoing, and recruiting clinical trials. Supplementary searches of official regulatory databases, including the United States Food and Drug Administration (U.S. FDA), European Medicines Agency (EMA), and United Kingdom Medicines and Healthcare products Regulatory Agency (MHRA), were performed to verify regulatory information. The search strategy did not impose a lower publication-date threshold, capturing all records from database inception through 31 July 2026, which was defined *a priori* as the evidence cutoff date. Search strategies combined controlled vocabulary (where available) with free-text keywords including ‘CRISPR-Cas9,’ ‘genome editing,’ ‘gene editing,’ ‘base editing,’ ‘prime editing,’ ‘epigenome editing,’ ‘delivery systems,’ ‘cancer,’ ‘oncology,’ ‘hematological disorders,’ ‘inherited genetic diseases,’ ‘clinical trials,’ ‘gene therapy,’ ‘genomic safety,’ ‘off-target effects,’ and ‘clinical translation.’ Boolean operators (AND/OR) were applied to optimize retrieval of relevant publications. The complete database-specific search strategies, including the full PubMed search string, targeted supplementary searches, and source-specific search approaches, are provided in Supplementary Table S1.

### 2.2 Study selection

Titles and abstracts retrieved from the database searches were screened for relevance to the scope of this review. Full-text articles were subsequently evaluated for relevance to therapeutic genome editing strategies, clinical translation, delivery technologies, genome editing safety, or regulatory and ethical aspects of CRISPR-Cas9. Clinical trial information was verified using publicly available clinical trial registries where applicable. Reference lists of eligible publications were manually screened to identify additional relevant studies. When multiple reports described the same clinical trial, priority was given to the most recent peer-reviewed publication reporting the largest patient cohort and longest available follow-up. Clinical trial characteristics and regulatory status were cross-verified using ClinicalTrials.gov, official regulatory documents, and corresponding primary publications whenever available. For evidence synthesis, retrieved publications were categorized into preclinical studies (cell culture and animal investigations), clinical studies (Phase I-IV clinical trials, where applicable), systematic reviews and meta-analyses, consensus guidelines, regulatory documents, and landmark methodological studies describing genome editing technologies. This evidence classification was used consistently throughout the review to distinguish experimental observations from clinically established evidence.

### 2.3 Inclusion criteria and study prioritization

Eligible evidence included peer-reviewed original research articles, clinical trial reports, systematic reviews, meta-analyses, consensus guidelines, and official regulatory documents relevant to therapeutic genome editing. Conference abstracts, editorials, letters, duplicate reports, and non-peer-reviewed preprints were excluded unless they contained unique clinical information unavailable elsewhere. Priority was given to primary clinical publications, official regulatory documents, and studies reporting therapeutic applications, long-term safety, genome editing outcomes, and clinical translation. To maximize the reliability of evidence synthesis, clinical outcomes, safety data, regulatory approvals, and trial characteristics were verified against primary sources whenever available, including original peer-reviewed clinical trial publications, official trial registry records (ClinicalTrials.gov and WHO ICTRP), and regulatory agency documents issued by the U.S. FDA, EMA, and MHRA. Secondary reviews were used primarily for background information and were cross-checked against corresponding primary sources before inclusion. When multiple publications described the same clinical trial, preference was given to the most recent peer-reviewed report with the largest patient cohort and the longest available clinical follow-up. Earlier reports describing interim analyses were consulted only when they contained unique information not available in subsequent publications. Duplicate reports were excluded to avoid overrepresentation of individual clinical studies.

## 3 Overview of CRISPR-Cas9 therapeutic modalities

### 3.1 *Ex vivo* versus *in vivo* genome editing

Therapeutic genome editing strategies are fundamentally categorized by the site of cellular modification: *ex vivo* and *in vivo*. The *ex vivo* approach involves harvesting patient-derived cells (e.g., hematopoietic stem and progenitor cells [HSPCs] or T cells), executing targeted genetic modification under controlled laboratory conditions, and subsequently reinfusing the edited cell product into the patient (Vavassori et al., 2023). In this scenario, cells are expanded and genetically modified using established *ex vivo* protocols with plasmid DNA, mRNA, or preassembled RNPs. *Ex vivo* manufacturing permits extensive pre-infusion quality control assessment, including the evaluation of on-target editing efficiency, cell viability, immunophenotypic identity, sterility, and structural genomic integrity. However, contemporary quality control assays evaluate representative cell fractions rather than the entire therapeutic product and therefore cannot guarantee the absolute absence of unintended sequence-level or structural genomic alterations (Hirakawa et al., 2020). Furthermore, safety profiling remains constrained by sampling limitations and assay limits of detection for ultra-low-frequency editing events. Although genome-wide off-target assays, targeted deep sequencing, optical genome mapping, and karyotypic analyses substantially improve safety evaluation, rare structural variants, large deletions, chromosomal translocations, and complex rearrangements may escape detection. These limitations are especially critical in polyclonal cell therapies, where the clonal behavior and oncogenic potential of rare aberrant subpopulations remain challenging to predict. Consequently, *ex vivo* editing significantly enhances pre-infusion safety profiling, but does not completely eliminate the risk of administering rare genomic aberrations (Lee et al., 2021).

In contrast, *in vivo* genome editing requires the direct systemic or local administration of CRISPR components to target tissues, such as the liver, central nervous system, or ocular compartments. *In vivo* delivery relies on both viral (e.g., adeno-associated viruses [AAVs]) and non-viral delivery platforms (e.g., lipid nanoparticles [LNPs] and virus-like particles). While *in vivo* delivery circumvents the costly and logistically demanding cell manufacturing pipelines of *ex vivo* approaches, it presents formidable challenges regarding tissue biodistribution, delivery efficiency, and host immunogenicity (Taha et al., 2022). Viral-vector delivery can achieve efficient *in vivo* transduction; however, DNA-based vectors that sustain Cas nuclease expression, particularly AAV-based systems, may prolong the window for unintended editing and can elicit immune responses against vector or editor components. Conversely, non-viral delivery platforms afford transient nuclease exposure, mitigating off-target risks, yet achieving functional extrahepatic delivery across diverse tissues remains a major translational bottleneck (Wei et al., 2020). For instance, LNP-mediated delivery of Cas9 mRNA and sgRNA provides a relatively transient window of nuclease activity, which may reduce the opportunity for cumulative off-target cleavage compared with prolonged editor expression (Wilson and Gilbert, 2018).

**FIGURE 3:**
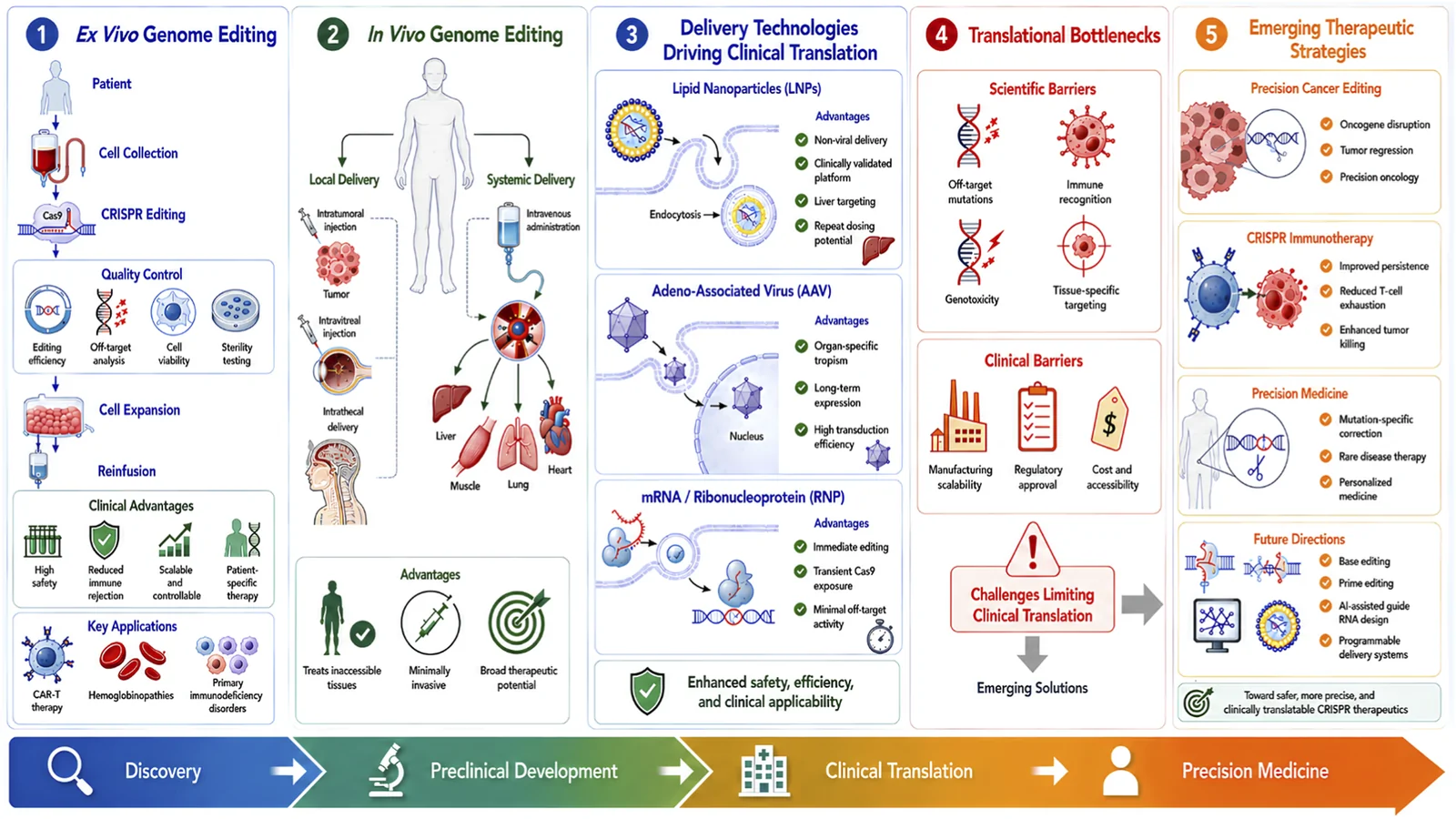
*Ex Vivo* and *In Vivo* Delivery Platforms for CRISPR-Cas9 Therapeutics. Schematic illustration of *ex vivo* and *in vivo* CRISPR-Cas9 genome editing strategies, highlighting major delivery technologies (lipid nanoparticles, adeno-associated viruses, and mRNA/ribonucleoprotein systems), key translational bottlenecks (scientific and clinical barriers), emerging therapeutic strategies, and the translational pathway from discovery to precision medicine.

Overall, *ex vivo* and *in vivo* modalities possess distinct translational advantages and constraints. *Ex vivo* editing provides rigorous control over editing parameters, cell dosing, and safety screening, making it the most clinically mature genome editing approach for hematologic and selected immune cell applications. Conversely, *in vivo* editing broadens therapeutic applicability to anatomically restricted or non-harvestable tissues, but remains governed by the biodistribution of delivery vectors, on-target efficiency, and systemic immunogenicity. Consequently, the choice of editing modality depends on the underlying disease biology, target tissue accessibility, and the balance between therapeutic efficacy and safety. Figure 3 illustrates major *ex vivo* and *in vivo* genome editing strategies, along with key translational bottlenecks and emerging therapeutic strategies leading to clinical translation.

### 3.2 Editing strategies and outcome types

Although CRISPR-Cas9, base editing, prime editing, epigenome editing, and gene addition all enable therapeutic genome manipulation, they differ substantially in their editing mechanisms, precision, dependence on repair pathways, delivery requirements, and clinical maturity. Consequently, selection of the most appropriate platform depends on the underlying mutation, disease biology, target tissue, and acceptable risk profile (Newman and Ausubel, 2016). Using the error-prone NHEJ pathway to introduce insertions or deletions (indels) at the cleavage site disrupts pathogenic alleles or oncogenes, thereby achieving targeted gene disruption. The CRISPR-STOP strategy extends this knockout approach by using base editors to convert specific codons into premature termination codons (PTCs) without intentionally generating a DSB, thereby mitigating DSB-associated structural variations while retaining base-editor-specific risks such as bystander and off-target deamination. These strategies have demonstrated therapeutic efficacy in preclinical models by disrupting genes encoding key viral co-receptors or oncogenic drivers (Wang and Doudna, 2023). HDR-based strategies use an exogenous donor template to achieve precise sequence correction or targeted sequence insertion (Ishino et al., 2018). Classical donor-templated HDR is most active in S/G2 and is inefficient in many quiescent or terminally differentiated cells, although alternative targeted-integration strategies are under development. Base editors and prime editors overcome several limitations of conventional nuclease-mediated editing by avoiding reliance on HDR (Lee et al., 2025). However, each platform possesses distinct limitations. Cytosine and adenine base editors may generate bystander nucleotide substitutions within the editing window and gRNA-independent off-target deamination (Shmuel-Eidelman et al., 2026). Prime editing generally offers broader editing capabilities and reduced bystander editing but often has lower editing efficiency, requires larger delivery cargo, and may produce unintended editing products (Chen and Liu, 2023). Consequently, the relative advantages of each platform remain highly context-dependent rather than universally superior (Zhao et al., 2023). Lastly, epigenome modulation offers a cleavage-independent approach by using dCas9 to recruit transcriptional activators or repressors to promoters and enhancers. This approach enables potentially reversible regulation of gene expression, thereby offering a way to silence harmful transcripts or upregulate compensating genes without changing the underlying nucleotide sequence; however, the duration and reversibility of the induced chromatin state are locus- and effector-dependent. Advanced dCas9-based epigenome editors fused to chromatin-modifying enzymes, such as p300 or HDAC3, further extend this capability by enabling the targeted modulation of regulatory elements to durably alter cellular phenotypes (Azangou-Khyavy et al., 2020).

### 3.3 Comparative assessment of genome editing platforms

Although recent advances have substantially expanded the genome editing toolbox, no single platform is universally applicable to all therapeutic applications. These platforms are complementary; selection should be guided by the pathogenic variant, therapeutic objective, target-cell biology, delivery constraints, editing mechanism, and product-specific safety profile.

Conventional CRISPR-Cas9 remains the most clinically advanced platform and is particularly well suited for gene disruption strategies, multiplex genome engineering, and applications in which permanent inactivation of pathogenic genes is desirable, such as *BCL11A* enhancer disruption in hemoglobinopathies or immune-cell engineering for cancer immunotherapy (Psatha et al., 2018). However, because therapeutic efficacy depends on the generation of DSBs, the potential for structural genomic alterations must be carefully considered, particularly *in vivo* (Wang et al., 2025).

In contrast, base editing and prime editing are generally preferable when precise correction of pathogenic variants is required without generating DSBs. Base editing is especially suitable for transition mutations that constitute a large proportion of pathogenic single-nucleotide variants, whereas prime editing offers greater flexibility for introducing small insertions, deletions, and a broader range of nucleotide substitutions that are not addressable by current base editors, without depending on DSB formation (Aliciaslan et al., 2026). Nevertheless, the broader editing scope of prime editing is currently balanced against lower editing efficiency, larger delivery cargo, and greater manufacturing complexity (Lushington et al., 2026). It is important to clarify that p53 pathway activation and complex structural rearrangements such as chromothripsis are safety liabilities associated with DSB-dependent editing (most notably conventional CRISPR-Cas9), not capabilities or features of prime editing (Haapaniemi et al., 2018; Ihry et al., 2018; Aussel et al., 2025). Although prime editing avoids an intentional DSB and may reduce DSB-associated structural alterations, product-specific risks include unintended indels, pegRNA-scaffold incorporation, incomplete editing products, and nick-associated off-target effects, particularly with PE3-type designs (Anzalone et al., 2020).

Epigenome editing is a distinct therapeutic strategy that modulates gene expression without altering the underlying DNA sequence. This strategy may be advantageous when transient or reversible regulation of disease-associated genes is sufficient, particularly for disorders in which permanent genomic modification may be unnecessary or undesirable (Yuan et al., 2026). Likewise, gene-addition approaches remain valuable when endogenous gene correction is technically impractical, such as disorders involving large deletions or complete loss of gene function (Maeder and Gersbach, 2016).

As genome editing technologies continue to mature, future therapeutic development will likely rely on selecting the most appropriate editing modality based on the mutation spectrum, target-cell biology, delivery constraints, and long-term safety considerations rather than attempting to apply a single editing platform across all disease settings. This comparative framework highlights that these technologies should be regarded as complementary therapeutic approaches whose clinical utility depends on the disease’s biological context. Selection should be guided by mutation type, therapeutic objective, target-cell biology, delivery feasibility, and product-specific safety considerations. Table 1 presents a comparative overview of the principal characteristics of the major therapeutic genome editing platforms.

**TABLE 1:** Comparative assessment of therapeutic genome editing and gene-addition platforms.

Clinical consideration	Conventional CRISPR-Cas9	Base editing	Prime editing	Epigenome editing	Gene Addition	References
Preferred mutation type	Gene knockout, regulatory element disruption, or targeted deletion	Transition SNVs	Transition and transversion SNVs, small indels	Gene dysregulation	Large deletions or absent gene expression	(Anzalone et al., 2020)
Best therapeutic objective	Gene disruption	Precise nucleotide correction	Precise sequence replacement	Gene activation/repression	Functional gene restoration	(Musunuru et al., 2021)
Suitable for multiplex editing	Well established, but multiple DSBs increase translocation/rearrangement risk	Moderate	Multiplexing demonstrated but limited by cargo, efficiency, and pegRNA design	Multiplexable, with locus- and effector-dependent regulation	Limited	(Wang et al., 2020a)
Dependence on HDR	No for disruption; generally yes for classical donor-template precise knock-in	No	No	No	Not applicable; depends on episomal expression, vector integration, transposase/integrase activity, or targeted-insertion design	(Yeh et al., 2019)
Suitability for non-dividing cells	Limited for HDR-based correction	Good	Good	Excellent	Good	(Newby et al., 2021)
DSB dependency	Yes (obligate DSB)	No (deaminase-mediated, nick-based)	No (nick-based, RT-mediated)	No (catalytically dead Cas9)	Not inherently DSB dependent; gene addition may be episomal, randomly integrating, transposase/integrase mediated, or coupled to targeted nuclease-assisted insertion	(Yeh et al., 2019; Anzalone et al., 2020)
Relative off-target/genotoxicity risk	Moderate-high (DSB-associated indels, translocations, chromothripsis)	Low-moderate (bystander/off-target deamination)	Lower DSB-associated structural-variant risk; pegRNA-, nick-, and editor-associated unintended products remain possible; long-term clinical safety is still being established	No DSB-associated genotoxicity from the intended mechanism; off-target transcriptional or epigenetic modulation remains possible	Variable (integration-site dependent for integrating vectors)	(Doench et al., 2016; Tsai et al., 2017; Aussel et al., 2025)
Relative editing efficiency (current generation)	High	Moderate-high	Low-moderate	Variable (locus/context dependent)	High (established gene-addition platforms)	(Kosicki et al., 2018b; Anzalone et al., 2020)
Delivery cargo requirement	SpCas9 coding sequence is ∼4.1 kb; promoters, guide cassette, regulatory elements, and vector sequences increase the total package beyond the single-AAV capacity	Moderate-large (Cas9-deaminase fusion)	Large (Cas9 nickase-RT fusion + pegRNA)	Moderate-large (dCas9-effector fusion)	Large (full transgene cassette; vector-dependent)	(Anzalone et al., 2020; Lushington et al., 2026)
Manufacturing complexity	Low-moderate (established)	Moderate	High	Moderate	Moderate-high (vector-dependent, but GMP-established)	(Lushington et al., 2026)
Current clinical maturity	Highest	Early clinical translation	Early clinical translation	Predominantly preclinical	Clinically established (gene therapy)	(Gillmore et al., 2021)
Major translational challenge	DSB-associated genotoxicity	Bystander editing	Delivery complexity and efficiency	Durability of regulation	Vector-related limitations	(High and Roncarolo, 2019)
Representative therapeutic applications	Hemoglobinopathies, CAR-T	Monogenic SNVs	Precise mutation correction	Transcriptional disorders	Large gene deficiencies	(Frangoul et al., 2021)

### 3.4 Delivery systems for CRISPR therapeutics

Efficient intracellular delivery of CRISPR components, including Cas nucleases (e.g., Cas9) and gRNAs, is a prerequisite for successful therapeutic genome editing (Chehelgerdi et al., 2024). Adeno-associated viral (AAV) vectors remain among the most widely investigated delivery platforms due to their efficient transduction of both dividing and non-dividing cells, broad spectrum of tissue-specific serotypes, and predominantly episomal persistence (Maeder and Gersbach, 2016). A major limitation of AAV vectors is their limited cargo capacity (∼4.7 kb); however, additional challenges include pre-existing and treatment-induced humoral immunity, neutralizing antibodies that may prevent repeat administration, prolonged nuclease expression that may increase cumulative off-target editing, dose-dependent toxicities, manufacturing complexity, variable biodistribution, and the potential for vector capture or rare genomic integration at CRISPR-induced DNA break sites (Colella et al., 2018). Consequently, vector selection requires balancing delivery efficiency against long-term safety considerations. Lentiviruses provide approximately 8 kb of capacity, infect both dividing and non-dividing cells, and integrate into the host genome, potentially resulting in insertional oncogenesis (Sinclair et al., 2023).

Helper-dependent adenoviral vectors can accommodate approximately 30–36 kb, whereas earlier-generation adenoviral vectors have lower usable capacities. Adenoviral genomes remain predominantly episomal, but strong innate and adaptive immune responses constrain systemic use (Rosewell and Vetrini, 2011; Ricobaraza et al., 2020). Non-viral delivery systems generally exhibit lower risks of insertional mutagenesis and prolonged nuclease expression than viral vectors and may reduce certain immunogenicity-related concerns. However, their overall safety profile depends on factors such as formulation, target tissue, dosing strategy, and therapeutic context, and therefore should not be considered universally safer than viral delivery systems (Cheng et al., 2021). LNPs are among the most extensively investigated non-viral delivery platforms for therapeutic CRISPR applications. They encapsulate CRISPR cargo, such as plasmid DNA, mRNA encoding Cas9, or RNP complexes. Optimization of lipid composition, particle size, and surface charge improves cellular uptake, endosomal escape, and delivery efficiency (Wang et al., 2024). CRISPR-Cas9-based LNPs have targeted the transthyretin (*TTR*) gene in hepatocytes, thereby decreasing toxic protein production (Gillmore et al., 2021). Although LNP-mediated delivery provides transient nuclease expression and avoids vector integration, repeat administration is not universally feasible. Repeated dosing depends on lipid composition, target tissue, immune activation, cumulative toxicity, and formulation-specific pharmacokinetics. Consequently, the suitability of repeat administration should be evaluated individually for each therapeutic platform rather than considered an inherent advantage of LNP delivery (Brimacombe et al., 2025). Polymeric nanoparticles are versatile, non-viral carriers designed to respond to microenvironmental signals, such as pH variations, and release genome editing cargo in a targeted manner (Beach et al., 2024). RNP delivery involves introducing preassembled Cas9-gRNA RNP complexes directly into target cells (DeWitt et al., 2017). This approach offers several advantages: it avoids introducing a DNA expression cassette, limits editor exposure, and therefore reduces the opportunity for persistent nuclease expression or capture of vector/plasmid DNA at cleavage sites. Physical approaches such as electroporation and microinjection are particularly effective for delivering CRISPR components into cells and can be used for *ex vivo* or local delivery.

GalNAc conjugation enables hepatocyte-selective uptake of compatible oligonucleotide cargo through the asialoglycoprotein receptor; delivery of a complete genome editor generally requires additional cargo and formulation strategies (Zhang et al., 2025). In cancer treatment, delivery strategies exploit characteristic features of the tumor microenvironment (TME). For example, nanocarriers may respond to acidic pH or elevated protease activity (e.g., matrix metalloproteinases) in the TME, thereby enabling selective release of CRISPR cargo within the tumor (He et al., 2024). Active targeting can be accomplished by antibody- or peptide-conjugated nanoparticles that bind tumor-specific antigens, such as HER2, and are preferentially delivered to cancer cells (Rauf et al., 2025). Extracellular vesicles (EVs), such as exosomes, are considered endogenous nanocarriers for targeted delivery of CRISPR components due to their biocompatibility and ability to shuttle cargo between cells (Yan and Liang, 2022). Although viral vectors generally provide higher transduction efficiency and sustained gene expression, their clinical application is constrained by limited cargo capacity, immunogenicity, and prolonged nuclease expression that may increase the risk of unintended genome editing. Conversely, non-viral delivery systems may reduce certain risks associated with prolonged nuclease expression and insertional mutagenesis while providing transient CRISPR activity and greater manufacturing flexibility. However, their overall safety and clinical suitability depend on the specific delivery platform and therapeutic application, and they currently exhibit lower delivery efficiency in several extrahepatic tissues. Therefore, no single delivery platform is universally superior. Selection of an appropriate delivery platform should be guided by disease biology, tissue accessibility, cargo requirements, desired duration of editor expression, manufacturing feasibility, and the overall balance between efficacy and safety for the intended clinical application. The principal viral and non-viral delivery systems employed for therapeutic CRISPR-Cas9 applications differ considerably in their cargo capacity, delivery efficiency, tissue tropism, and translational limitations. Table 2 summarizes their key characteristics.

**TABLE 2:** Comparative characteristics of major viral and non-viral delivery systems for CRISPR-Cas9 therapeutics.

Delivery Platform	Cargo Delivered	Cargo capacity	Duration of editor expression	Target tissue/Tropism	Repeat Dosing Feasibility	Immunogenicity	Integration Risk	Major Advantages	Principal Limitations	Current clinical Maturity	References
Adeno-associated virus (AAV)	DNA encoding Cas9 and sgRNA	Limited (∼4.7 kb)	Long-term (episomal expression)	Broad; serotype-dependent, efficient in liver, retina, CNS and muscle	Limited due to pre-existing and treatment-induced neutralizing antibodies	Moderate; serotype-dependent	Rare vector integration reported, particularly at CRISPR-induced DSBs	High *in vivo* transduction efficiency, tissue-specific serotypes, clinically well established	Small cargo capacity, prolonged nuclease expression, repeat-dosing limitations, dose-related toxicity, manufacturing complexity	High as a gene-therapy vector; early clinical experience for *in vivo* CRISPR delivery	(Madigan et al., 2023)
Lentiviral vector (LV)	DNA encoding Cas9/sgRNA	Large (∼8–10 kb)	Stable, long-term expression	Primarily *ex vivo* delivery to HSPCs and T cells	Generally not applicable for repeated *in vivo* administration	Moderate	High (integrating vector)	Efficient transduction of dividing and non-dividing cells, large cargo capacity	Insertional mutagenesis, persistent nuclease expression, biosafety concerns	High (mainly *ex vivo*)	(Milone and O’Doherty, 2018)
Adenoviral vector (AdV)	DNA encoding Cas9/sgRNA	Very large (>30 kb)	Transient	Broad tissue tropism	Restricted due to potent innate and adaptive immune responses	High	Very low; predominantly non-integrating	Very large cargo capacity, high transduction efficiency	Strong innate and adaptive immune responses, inflammatory toxicity	Established as a vector class, but CRISPR-editor delivery remains mainly preclinical/early translational	(Riedl et al., 2022)
Lipid nanoparticles (LNPs)	Cas9 mRNA + sgRNA or RNP	Moderate to large	Transient	Predominantly liver; expanding to extrahepatic tissues	Potentially feasible, formulation dependent	Low-moderate	None	Transient editor expression, no vector integration, scalable manufacturing	Limited tissue specificity, lower efficiency outside liver, formulation-dependent repeat dosing, lipid-associated toxicity	Clinical proof-of-concept established for hepatic *in vivo* editing; late-stage clinical development ongoing	(Duan et al., 2021)
Polymeric nanoparticles	DNA, mRNA or RNP	Flexible	Transient	Variable; formulation dependent	Potentially feasible	Low	None	Biodegradable, customizable surface chemistry, low immunogenicity	Lower transfection efficiency, limited clinical experience	Early clinical/preclinical	(Duan et al., 2021)
Gold nanoparticles	RNP, DNA or oligonucleotides	Moderate	Transient	Localized delivery	Potentially feasible	Generally low/preclinically favorable; formulation/source dependent	None	Precise local delivery, minimal immunogenicity	Limited systemic delivery, manufacturing cost	Preclinical	(Lee et al., 2017)
Cell-penetrating peptides (CPPs)	RNP or protein complexes	Limited	Very transient	Local delivery	Feasible	Generally low/preclinically favorable; formulation/source dependent	None	No viral vector, minimal genomic persistence	Poor stability, limited intracellular delivery efficiency	Preclinical	(Ramakrishna et al., 2014)
Extracellular vesicles (EVs)/Exosomes	Cas9 mRNA, sgRNA or RNP	Moderate	Transient	Natural cell-derived targeting	Potentially feasible	Generally low/preclinically favorable; formulation/source dependent. Potential donor-cell cargo, heterogeneity, purification, and innate/adaptive immune considerations	None	Excellent biocompatibility, low immunogenicity, natural delivery vehicle	Low loading efficiency, manufacturing and purification challenges	Early preclinical	(Zhu et al., 2023)
Electroporation (*ex vivo*)	Cas9 RNP or mRNA	Not limited	Transient	*Ex vivo* only	Not applicable	None	None	High and reproducible *ex vivo* delivery efficiency for many HSPC and T cell workflows	Cell toxicity, unsuitable for direct *in vivo* delivery	Established for manufacturing several clinically advanced *ex vivo* genome-edited cell products	(Roth et al., 2018)

## 4 Therapeutic applications in cancer

### 4.1 Targeting oncogenic drivers and tumor suppressors

CRISPR-based genome editing has emerged as a versatile platform for investigating cancer biology and developing targeted therapeutic strategies. Cancer arises through the accumulation of genetic and epigenetic alterations that activate oncogenes and inactivate tumor suppressor genes (Anbarasu et al., 2025; Anbarasu and Anbarasu, 2026; Kedari et al., 2026). CRISPR-Cas9 enables targeted genome modification of these genetic alterations, thereby facilitating mechanistic studies and therapeutic development (Balon et al., 2022). Preclinical studies have demonstrated efficient CRISPR-Cas9-mediated disruption of oncogenic drivers such as *KRAS* and *EGFR*, although these approaches have not yet progressed to routine clinical application. Preclinical studies have also explored CRISPR-based manipulation of tumor-suppressor pathways, including *TP53* and *PTEN*; however, therapeutic restoration of tumor-suppressor function remains substantially more challenging than gene-disruption strategies and has not entered routine clinical use (Rehman and Abbas, 2026). Preclinical studies have also explored restoration of *PTEN* expression as an anticancer strategy, although clinical evidence remains limited. Fusion oncogenes (FOs) represent attractive therapeutic targets because they are frequently tumor-specific and are often required for malignant cell survival. Preclinical studies have demonstrated selective elimination of cancer cells through CRISPR-Cas9-mediated targeting of FOs, resulting in substantial tumor regression in mouse models (Martinez-Lage et al., 2020). Although these preclinical findings demonstrate the versatility of CRISPR-mediated targeting of oncogenic pathways, directly correcting cancer genomes remains substantially more challenging than genome editing for monogenic disorders. Unlike inherited diseases that are often caused by a single pathogenic mutation, most cancers exhibit extensive intratumoral heterogeneity, ongoing clonal evolution, and multiple cooperating driver mutations. Consequently, editing a single oncogenic target may not eliminate malignant cell populations or prevent disease recurrence. As a result, current clinical development has largely focused on *ex vivo* engineering of immune cells rather than directly editing heterogeneous tumor genomes *in vivo*.

Beyond direct genome editing, CRISPR can increase tumor sensitivity to traditional cancer therapies by editing genes that encode drug resistance or DNA repair pathways, thereby increasing susceptibility to chemotherapy, radiotherapy, and targeted therapeutic agents. For example, silencing genes involved in repairing DNA damage, such as *PARP1* or *ATM*, makes cancer cells more vulnerable to DNA-damaging agents. Inhibition of anti-apoptotic genes also increases the sensitivity of tumor cells to cytotoxic drugs. Furthermore, CRISPR has the potential to overcome resistance to targeted therapy by editing genes that confer drug resistance, thereby resensitizing tumors to drugs that initially failed to act (Saber et al., 2020). Collectively, these preclinical findings suggest that CRISPR technology provides a versatile platform for interrogating oncogenic pathways and developing precision cancer therapies. Nevertheless, most evidence supporting direct editing of oncogenic drivers currently originates from preclinical models. Translating these strategies into routine clinical practice will require overcoming challenges related to tumor heterogeneity, efficient *in vivo* delivery, editing specificity, and sustained therapeutic efficacy before they can be widely adopted.

### 4.2 CRISPR-enhanced cancer immunotherapy

#### 4.2.1 Engineering CAR-T and TCR-T cells

CRISPR-based engineering of immune cells has substantially expanded the capabilities of adoptive cellular immunotherapy (Rabaan et al., 2023). CRISPR technology enables precise genetic engineering of immune cells and modulation of the TME, thereby enhancing antitumor immunity (Feng et al., 2024). Most ongoing studies evaluating CRISPR-engineered Chimeric Antigen Receptor (CAR)-T and T Cell Receptor (TCR)-T cells remain Phase I or Phase I/II clinical trials primarily designed to establish safety, feasibility, and preliminary efficacy rather than definitive therapeutic benefit. Although CAR-T cell therapy has demonstrated therapeutic potential, particularly for hematological malignancies, it has limited efficacy in solid tumors due to the immunosuppressive TME and T cell exhaustion (Wang et al., 2023). CRISPR-Cas9 technology enables the specific manipulation of T cells, thereby enhancing their ability to eliminate tumor cells. CRISPR-Cas9 can disrupt genes encoding inhibitory receptors, including programmed cell death protein 1 (PD-1) and cytotoxic T lymphocyte-associated protein 4 (CTLA-4), thereby reducing T cell exhaustion. Preclinical studies indicate that these modifications enhance the survival and effectiveness of T cells against tumors (Freen-van Heeren, 2021).

In addition, CRISPR enables multiplex genome editing, allowing simultaneous modification of multiple genomic loci within the same therapeutic cell product. One approach involves knocking out the endogenous T cell receptor alpha constant (*TRAC*) locus to mitigate the risk of graft-versus-host disease (GVHD) in allogeneic CAR-T therapies. Concurrently, the CAR transgene can be targeted to the *TRAC* locus, simultaneously disrupting endogenous TCR expression and enabling more regulated CAR expression (Eyquem et al., 2017). Several initial-phase clinical trials are currently underway to determine the safety, feasibility, and preliminary clinical efficacy of CRISPR-edited T cells in cancer. The first-in-human clinical study initiated in 2016 used *ex vivo* CRISPR-Cas9-edited *PDCD1* knockout T cells to treat non-small cell lung cancer (Lu et al., 2020). Early-phase clinical trials are evaluating CRISPR-engineered immune-cell therapies across multiple hematologic malignancies and selected solid tumors, while most direct genome editing strategies targeting tumor cells remain at the preclinical stage. Among all oncology applications discussed to date, *ex vivo* T cell engineering is the most clinically advanced use of CRISPR technology. Unlike direct genome editing of tumor cells, *ex vivo* editing enables extensive quality control testing before reinfusion, minimizes systemic exposure to genome editing components, and provides greater control over editing efficiency and product consistency. Consequently, most ongoing clinical trials have prioritized immune-cell engineering, reflecting its comparatively lower translational risk and greater regulatory maturity.

#### 4.2.2 Editing tumor and microenvironmental targets

The TME has emerged as an important target for CRISPR-based therapeutic intervention. CRISPR-based functional genomic screens have enabled systematic identification of key immune escape mechanisms and resistance pathways in cancer. Genome-wide CRISPR screens have identified tumor-intrinsic determinants of sensitivity and resistance to immune-mediated killing, including components of interferon-γ signaling and antigen-presentation pathways (Manguso et al., 2017; Patel et al., 2017). Studying the roles of these genes may lead to new therapeutic approaches that improve the efficiency of immunotherapies. Beyond direct genome editing of tumor cells, CRISPR can also be used to reprogram the TME, which often hinders effective antitumor immunity (Huang et al., 2020). The TME consists of cellular and non-cellular elements that can promote or inhibit immune responses. For example, genetically modifying stromal cells to attenuate the secretion of transforming growth factor-beta (TGF-β) can reprogram the TME into a robust immunostimulatory state (de Streel and Lucas, 2021).

One notable example is CRISPR-Cas9-mediated editing of the *GDF15* gene in hepatocellular carcinoma (HCC) cells. Preclinical studies suggest that *GDF15*-targeted genome editing strategies may modulate the immunosuppressive TME and induce immune cell-mediated killing of HCC cells in mouse models (Du and Zhao, 2024; He et al., 2024). These findings suggest that simultaneous targeting of tumor cells and the surrounding microenvironment may enhance therapeutic efficacy. Such dual-targeting strategies may provide mechanistic insights into tumor–immune interactions while improving therapeutic responses (Anbarasu and Anbarasu, 2023; Chintala et al., 2023).

### 4.3 Oncolytic and virotherapeutic applications

Oncolytic viruses (OVs) are naturally occurring or engineered viruses that preferentially replicate within and lyse tumor cells while limiting, rather than completely excluding, damage to normal tissues. These intrinsic biological properties render OVs attractive therapeutic candidates, whose efficacy can be further potentiated through CRISPR-Cas engineering. The CRISPR system can be used to develop OVs that are more tumor-specific, optimize viral replication within tumor cells, and induce stronger immune responses, thereby enhancing their antitumor activity. Moreover, OVs may be modified with CRISPR to produce different immunomodulatory molecules or sensitized to interferon, thereby further increasing their effectiveness against malignant cells (Xiao et al., 2026). For example, oncolytic adenoviruses may be engineered to include a CRISPR-Cas9 system to strategically disrupt *CD274* (encoding PD-L1), thereby reducing PD-L1 expression in infected tumor cells. This dual-action system can not only exert a potent oncolytic effect but also neutralize a critical immune checkpoint, thereby significantly enhancing antitumor immune responses. Preclinical studies have also investigated CAR-T cells as carriers for systemic delivery of oncolytic HSV to solid tumors (Zhang Z. et al., 2024).

For virus-associated malignancies, including those linked to human papillomavirus (HPV), hepatitis B virus (HBV), or Epstein-Barr virus (EBV), the CRISPR systems have been investigated preclinically to disrupt viral oncogenes or other viral genetic elements (Xiao et al., 2025). The relevant viral genomic state is virus-dependent: HPV and HBV sequences may integrate into host DNA, whereas EBV genomes generally persist predominantly as episomes. These approaches remain predominantly preclinical and require substantial additional validation before clinical efficacy can be inferred. The use of CRISPR technology in oncolytic virotherapy is an important area of investigation in cancer therapy development.

### 4.4 *In vivo* CRISPR-Cas9 strategies for solid tumors

CRISPR-Cas9 genome editing *in vivo* is a potential strategy for directly modifying tumor cells without complex *ex vivo* cell manipulation. Successful *in vivo* editing largely depends on the development of efficient delivery systems that deliver CRISPR components to tumor tissues while minimizing systemic toxicity and off-target effects (Huang et al., 2022). Current *in vivo* CRISPR delivery methods generally fall into local and systemic delivery categories. Local delivery typically involves the direct application of CRISPR constructs to tumor tissues via intratumoral injection. This method achieves a high concentration of genome editing machinery at the tumor site, reducing exposure to non-target tissues. Intratumoral delivery has been widely used in preclinical models to target oncogenes or genes involved in tumor progression. For example, local CRISPR delivery in tumor-bearing mice disrupted oncogenic drivers, leading to reduced tumor growth and increased tumor cell apoptosis (Song et al., 2024).

Systemic delivery strategies aim to distribute CRISPR components throughout the body to reach metastatic tumors or tumors in inaccessible tissues. LNPs are among the most extensively investigated non-viral delivery systems for systemic CRISPR delivery. These nanoparticles can encapsulate Cas9 mRNA or RNP complexes along with gRNAs, enabling cellular uptake via endocytosis. Viral vectors, especially AAV, are also widely used for their high transduction efficiency and organ-specific targeting. AAV-mediated CRISPR delivery has demonstrated efficient genome editing across various organs and is being explored for solid-tumor targeting. Preclinical studies in animal models have demonstrated the therapeutic potential of *in vivo* CRISPR editing of solid tumors. CRISPR-mediated silencing of cancer-driving mutations in lung cancer mouse models resulted in reduced tumor burden and prolonged survival (Lei et al., 2023). Similarly, editing immunogenic genes enhanced antitumor immunity and limited tumor growth (Park et al., 2025). Despite these promising results, challenges such as delivery efficacy, immune responses to Cas proteins, and off-target effects remain critical for clinical translation. Despite encouraging preclinical activity, successful clinical translation of direct *in vivo* genome editing for solid tumors remains considerably more difficult than liver-targeted or *ex vivo* applications. Efficient tumor-specific delivery, heterogeneous antigen expression, limited penetration into the tumor microenvironment, and immune recognition of genome editing components continue to limit therapeutic effectiveness. Therefore, further advances in delivery technologies and tumor-selective targeting strategies will determine whether *in vivo* CRISPR editing becomes a broadly applicable treatment modality for solid malignancies.

### 4.5 Clinical trials of CRISPR-Cas9 in oncology

In the last few years, there has been substantial progress in the clinical translation of CRISPR-Cas9 genome editing technologies, and several clinical trials are examining their potential use in oncology. Most initial CRISPR-based cancer studies have involved *ex vivo* genome editing, in which immune cells are collected, genetically engineered using CRISPR technology, and reinfused to enhance anti-tumor immune activity (Rehman and Abbas, 2026). Such strategies enhance the efficacy of adoptive cell therapies by increasing cell persistence, tumor detection, and cytotoxicity (Shum et al., 2018). One of the earliest clinical applications of CRISPR in oncology was reported in 2016 for advanced non-small cell lung cancer, using patient-derived T cells to edit the *PDCD1* gene. Here, CRISPR-Cas9 was used to disrupt *PDCD1* in T cells, thereby reducing PD-1 expression, before they were reinfused into the patients. This approach was intended to prevent immune checkpoint-mediated suppression of anti-tumor immunity (Lu et al., 2020). The findings showed that CRISPR-edited T cells engrafted safely and persisted without dose-limiting toxicities. No objective responses were reported; the study primarily established manufacturing feasibility and short-term safety, with limited editing efficiency and persistence.

Subsequent clinical trials have investigated next-generation genome editing methods that incorporate multiplex CRISPR modifications to enhance the cell’s immune capabilities. In other studies, CRISPR has been used to knock out multiple genes that restrain T cell activity and, at the same time, insert tumor-specific receptors such as chimeric antigen receptors (CARs) or engineered tumor-specific TCRs. These modifications are intended to improve tumor recognition, reduce immunosuppression, and enhance the therapeutic efficacy of adoptive cell therapies for hematological malignancies and solid tumors (Qian and Liu, 2025). Currently, most CRISPR-based oncology clinical studies remain in Phase I or Phase I/II trials designed primarily to evaluate safety, feasibility, and preliminary clinical activity. Early clinical studies have not identified a consistent pattern of serious toxicity clearly attributable to unintended genome editing; however, small cohort sizes and limited follow-up duration preclude definitive conclusions regarding rare or delayed genotoxic events (Zhang et al., 2023). CRISPR-engineered T cells, NK cells, and HSCs are being tested in trials for cancers such as leukemia, lymphoma, melanoma, and refractory solid tumors (Chakraborty et al., 2026). Although early-phase clinical studies have established the feasibility and acceptable short-term safety of CRISPR-edited immune-cell therapies, evidence for durable clinical efficacy remains limited because most studies have involved small patient cohorts and early-phase trial designs. Furthermore, current clinical experience is dominated by *ex vivo* immune-cell engineering, whereas direct *in vivo* editing of solid tumors remains largely preclinical. Further clinical development will depend on improving delivery efficiency, minimizing unintended genomic alterations, overcoming tumor heterogeneity and immune escape, and developing scalable manufacturing processes to support broader clinical implementation.

Overall, the clinical translation of CRISPR-based oncology differs fundamentally from its application in inherited genetic disorders. Whereas correction of single-gene disorders seeks durable modification of relatively homogeneous cell populations, cancer therapy must contend with genetically diverse tumors, dynamic clonal evolution, and complex interactions within the tumor microenvironment. Consequently, *ex vivo* immune-cell engineering has emerged as the leading clinical application of CRISPR in oncology, while direct *in vivo* genome editing of solid tumors remains an important long-term translational objective. Continued improvements in delivery systems, multiplex editing strategies, and genome editing safety will be essential for expanding the clinical impact of CRISPR-based cancer therapeutics.

## 5 Therapeutic applications in inherited genetic diseases

### 5.1 Hematologic disorders

Hematologic disorders currently represent the most clinically advanced application of therapeutic CRISPR-Cas9 genome editing. Monogenic blood diseases are particularly amenable to correction using genome editing technology, since HSPCs can be harvested, edited *ex vivo*, and reinfused after myeloablative conditioning; less toxic conditioning approaches are under investigation, but current approved exa-cel treatment still requires full myeloablation (Ferrari et al., 2021). Sickle cell disease (SCD) and transfusion-dependent β-thalassemia (TDT) represent the most clinically advanced examples of therapeutic genome editing. Rather than correcting the β-globin (*HBB*) mutation itself, a leading clinically validated strategy for SCD and TDT involves CRISPR-Cas9 disruption of the erythroid-specific *BCL11A* enhancer. In SCD, fetal hemoglobin (HbF) inhibits HbS polymerization and reduces sickling; in TDT, increased γ-globin/HbF partially compensates for deficient β-globin production and ineffective erythropoiesis. Clinical studies have reported sustained HbF production, marked reductions in vaso-occlusive crises among patients with SCD, and durable transfusion independence in many treated patients with TDT during currently available follow-up (Frangoul et al., 2024; Locatelli et al., 2024).

These clinical outcomes contributed to the regulatory approval of the first CRISPR-based therapies for hemoglobinopathies. No treatment-related leukemogenesis or clinically concerning clonal dominance has been reported in the published follow-up to date; nevertheless, prolonged surveillance remains necessary because rare delayed events cannot yet be excluded (Lee et al., 2021). In addition to hemoglobinopathies, CRISPR technologies for inherited hematologic disorders, including gene correction and gene safe harbor integration, are currently being explored. However, despite significant advances in clinical practice, several challenges remain. The myeloablative conditioning regimen used to ablate endogenous HSCs/HSPCs carries toxic risks; additionally, efficient HDR-mediated correction of long-term repopulating HSCs remains technically challenging and continues to limit broader clinical application.

### 5.2 Ophthalmologic and neuromuscular diseases

*In vivo* genome editing has emerged as an important therapeutic strategy for ocular and selected neuromuscular disorders. The eye represents an attractive target for *in vivo* genome editing because of its anatomical accessibility, immune-privileged environment, and compartmentalized anatomy that limits systemic exposure (Choi et al., 2023). The first reported clinical application of *in vivo* CRISPR genome editing for an inherited disease was the EDIT-101 trial for *CEP290*-associated Leber congenital amaurosis type 10 (LCA10). The study specifically targeted the c.2991 + 1655 A>G deep-intronic pathogenic variant in intron 26 of *CEP290*. EDIT-101 uses AAV5-delivered SaCas9 and two gRNAs to excise the aberrant splice-generating region. The Phase I/II study reported an acceptable safety profile and preliminary improvements in visual function in a subset of treated patients (Pierce et al., 2024). Other inherited retinal dystrophies are being evaluated in preclinical models, including, but not limited to, those due to retinitis pigmentosa and Stargardt disease (Gómez-Escribano et al., 2025).

The limitations of AAV vectors for delivery and the size constraints of Cas proteins have prompted exploration of smaller Cas variants and dual-vector delivery systems (Kabadi et al., 2024). The systemic challenges posed by neuromuscular disorders like Duchenne muscular dystrophy (DMD) are even more pronounced than they are for other gene-based therapies. DMD is driven by pathogenic mutations in the dystrophin (*DMD*) gene. Conventional CRISPR-based therapeutic strategies have primarily focused on restoring the dystrophin reading frame through programmed exon deletion, splice-site editing, or other DNA-level strategies; distinguish these from antisense-oligonucleotide-mediated exon skipping at the RNA level (Choi and Koo, 2021). Preclinical data in animal models indicate that systemic AAV-mediated genome editing can partially restore dystrophin expression and improve muscle function. However, major translational challenges remain to successfully perform efficient genome editing in skeletal and cardiac muscle, including immune responses to the Cas proteins and to dystrophin; these challenges are still being studied (Haque and Yokota, 2025). Other neurogenetic disorders, including spinal muscular atrophy, have been investigated as potential targets for CRISPR-based therapies, but their clinical translation is limited by the ability of current CRISPR delivery systems to penetrate the blood-brain barrier and deliver editing tools to the central nervous system (CNS) (Salomonsson and Clelland, 2024). Although localized ocular genome editing has demonstrated early clinical feasibility, efficient multisystem genome editing for neuromuscular disorders remains considerably more challenging.

### 5.3 Metabolic and liver-targeted diseases

The liver is currently one of the most suitable target organs for *in vivo* CRISPR-based genome editing because it is readily accessible through systemic circulation and established nanoparticle delivery technologies. Efficient hepatocyte uptake following systemic administration of LNP-encapsulated CRISPR components has established the liver as one of the most clinically accessible organs for *in vivo* genome editing (Wu et al., 2026). One leading clinical example of LNP-mediated *in vivo* CRISPR therapy is transthyretin amyloidosis (ATTR). CRISPR-Cas9 editing of the *TTR* gene, delivered to hepatocytes via LNPs, has produced substantial reductions in circulating TTR concentrations after a one-time administration. Initial clinical trial data indicated that protein production could be decreased for an extended period without substantial adverse effects in patients receiving these treatments, supporting the preliminary clinical feasibility and acceptable short-term safety profile of *in vivo* CRISPR-mediated liver editing (Gillmore et al., 2021; 2025). Preclinical and early clinical programs use *in vivo* base editing to inactivate *PCSK9* in hepatocytes and lower low-density lipoprotein (LDL) cholesterol; efficacy, durability, and safety remain investigational (Chadwick et al., 2017; Vafai et al., 2026). CRISPR-based therapies for inherited metabolic disorders, including ornithine transcarbamylase (OTC) deficiency and phenylketonuria (PKU), remain in preclinical development, and use strategies that encompass both targeted gene correction and transgene insertion (Ijaz et al., 2025). Permanent edits can be inherited by daughter hepatocytes during cell division, but durable tissue-level benefit depends on hepatocyte turnover rates, the edited-cell fraction, and whether edited cells have a selective advantage (Zhang K. et al., 2024). However, long-term safety concerns related to off-target effects, potential unintended large DNA deletions, and potential immune responses following repeated dosing remain. CRISPR-based therapeutics aimed at gene modification in the liver represent an emerging therapeutic strategy toward broader systemic genome editing.

### 5.4 Other monogenic and complex inherited conditions

Beyond hematologic and hepatic diseases, CRISPR-Cas9 technologies are being explored for a range of monogenic disorders (Humbert et al., 2021). Cystic fibrosis (CF) is a genetic disorder caused primarily by mutations occurring in the *CFTR* gene. Two principal CRISPR-based therapeutic strategies are currently under investigation to correct the underlying genetic defect in CF: (i) an *ex vivo* approach using gene-corrected autologous airway basal stem cells followed by transplantation back into the patient; and (ii) an *in vivo* approach. Major biological and technical barriers limit the delivery of CRISPR components to airway epithelial cells, including the layered, pseudostratified architecture of the airway epithelium, which restricts vector access to basal progenitor cells (Vaidyanathan et al., 2022). Hemophilia A and B are also actively investigated targets for CRISPR-mediated genomic intervention; strategies could include the direct insertion of entire *F8* or *F9* genes into hepatocytes or insertion into a safe-harbor location resulting in stable production of functional clotting factors, and preclinical studies show encouraging results on restoration of coagulation activity (Zhang et al., 2019; Wang Q. et al., 2020). Preclinical investigations are currently evaluating inherited retinal disorders not caused by the *CEP290* gene, such as *RPE65*-associated dystrophy (Butt et al., 2025). At the same time, other neurogenetic disorders, such as Huntington’s Disease, are being investigated using allele-specific gene disruption to eliminate or reduce the expression of deleterious proteins (Shin et al., 2016).

**FIGURE 4:**
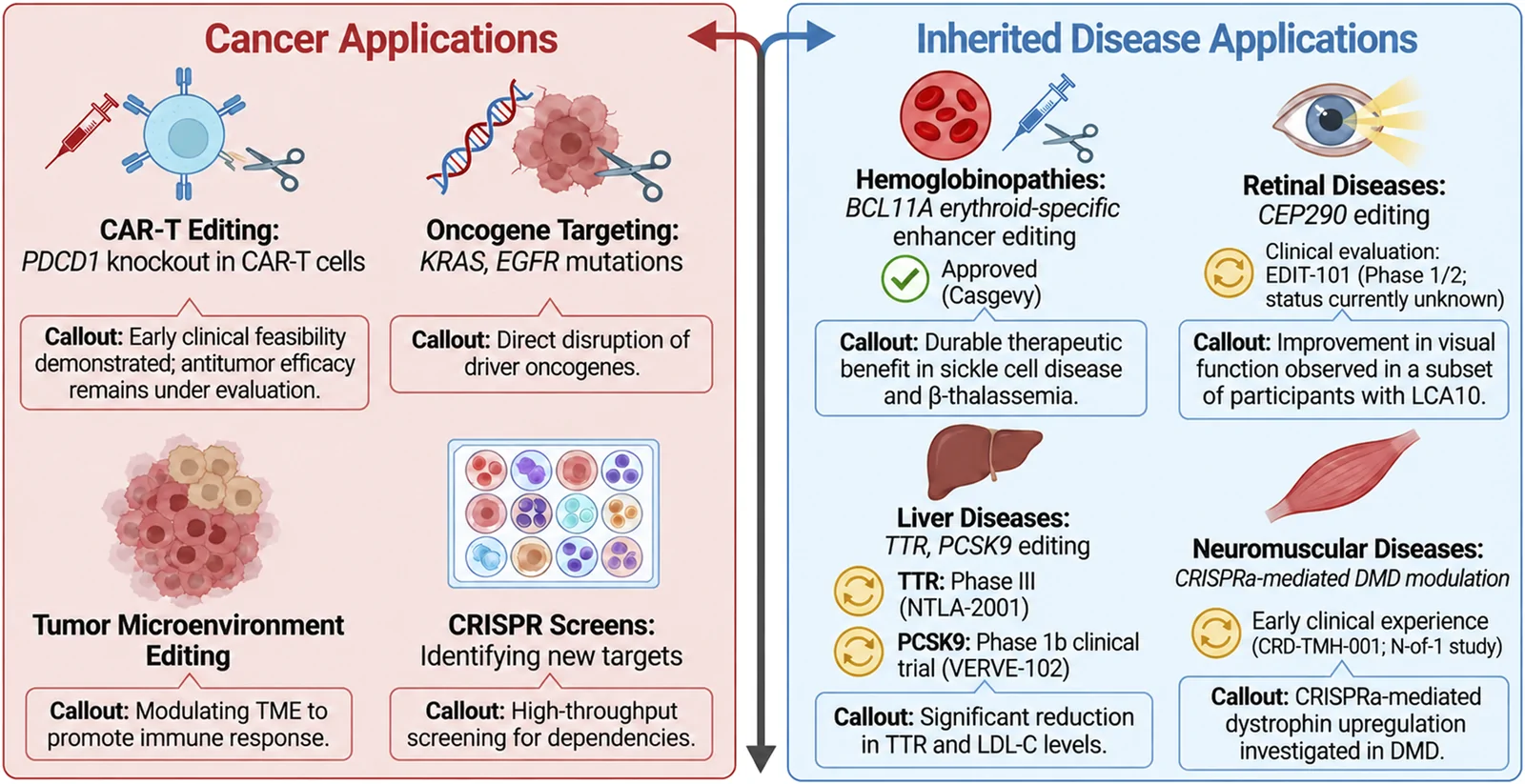
Therapeutic Applications of CRISPR-Cas9 in Cancer and Monogenic Diseases. In cancer, CRISPR enables oncogene targeting, preclinical tumor-suppressor restoration or modulation strategies, and immune cell engineering. In inherited diseases, it supports *ex vivo* and *in vivo* gene-correction strategies, including those for hemoglobinopathies, liver disorders, and retinal diseases. These applications highlight the expanding clinical potential of CRISPR-based precision medicine.

Polygenic and genetically complex disorders pose substantially greater challenges for therapeutic genome editing because disease pathogenesis is distributed across multiple loci and frequently involves multiple distinct tissues or cell types. Significant translational barriers for these systemic disorders include overcoming heterogeneous vector biodistribution to achieve efficient gene modification across all target tissues and securing a sufficient fraction of therapeutically edited cells (Kolanu, 2024; Qie et al., 2025). Furthermore, disorders that require precise gene correction rather than simple gene disruption may be better suited to a base- and/or prime-editing approach. Although most of the aforementioned applications are still in preclinical development, continued advances in delivery platforms and precision-editing technologies are expanding the spectrum of potentially treatable inherited disorders. Collectively, these studies highlight the expanding clinical landscape of CRISPR-Cas9 therapeutics across cancer and monogenic diseases (Figure 4).

### 5.5 Landscape of clinical trials for inherited diseases

Clinical development of CRISPR-based therapies for inherited diseases has expanded substantially over the past 5 years. Clinical trials have been broadly categorized by editing strategy (gene disruption versus correction), delivery method (*ex vivo* versus *in vivo*), and disease type. Clinical development has been led by *ex vivo* HSPC editing for SCD and TDT, culminating in regulatory approval of exagamglogene autotemcel, while multiple additional nuclease-, base-editing-, and prime-editing programs remain in early clinical development. Patients enrolled in early clinical studies of these therapies have demonstrated encouraging clinical outcomes with autologous transplantation, with planned long-term follow-up extending up to 10–15 years post-transplant to assess durability and genotoxic risks (Kerwash et al., 2024). *In vivo* investigations are increasingly focused on using CRISPR-Cas9 to treat liver and ocular diseases. Transthyretin amyloidosis represents one of the first *in vivo* CRISPR-Cas9 trials in humans conducted using LNP-mediated editing. Ocular trials for inherited retinal dystrophies utilize subretinal AAV delivery systems to achieve localized genome editing with immune privilege (Arbabi et al., 2019).

Clinical research using genome editing techniques for hereditary angioedema, hypercholesterolemia, and metabolic enzyme deficiencies is underway (Jalal et al., 2025). Studies utilize various editing strategies, including gene knockout strategies (e.g., *TTR* or *PCSK9* knockout) or targeted gene correction via HDR or next-generation editing approaches, including base and prime editing (Simoni et al., 2024). Common trends in clinical trial design include identifying safety endpoints, using molecular biomarker endpoints, and using clinically relevant functional endpoints. Regulatory agencies continue to require thorough genomic characterization of gene-edited cells before initiating clinical studies, as well as completion of a comprehensive biodistribution study and a longer-term follow-up evaluation to assess the possibility of delayed adverse events due to the genome editing procedure (Mizoguchi et al., 2024). Collectively, these clinical studies demonstrate continued expansion of early-stage clinical translation across multiple inherited genetic disorders while highlighting the need for longer-term efficacy and safety evaluation (Cetin et al., 2025). It also offers a wider range of therapeutic indications, demonstrating the gradual transition from early proof-of-concept studies toward broader clinical evaluation. Clinical studies have provided preliminary evidence of the feasibility, biological activity, and safety of CRISPR-based therapeutics across oncology and inherited genetic disorders, although the strength of clinical evidence varies substantially by indication, editing platform, study phase, and follow-up duration (Table 3). Importantly, the terms ‘molecular correction,’ ‘durable therapeutic benefit,’ ‘functional cure,’ and ‘clinical cure’ should not be used interchangeably when discussing therapeutic genome editing. Successful genomic editing does not necessarily translate into complete correction of all disease-causing alleles or universal clinical remission. Current CRISPR-based therapies primarily target somatic tissues and therefore aim to achieve durable therapeutic benefit rather than permanent disease eradication. Clinical maturity varies markedly by indication and modality. *Ex vivo* HSPC editing is established for hemoglobinopathies, whereas most *in vivo* programs remain investigational; durable benefit and delayed risks require indication-specific long-term follow-up.

**TABLE 3:** Representative clinical trials of CRISPR-based therapeutics in cancer and inherited genetic disorders (information cutoff date: 31 July 2026)*.

Trial Registry ID(s)	Disease/Indication	Therapeutic candidate (INN/Brand)	Target gene/Edit	Editing Modality	Trial Phase (at cutoff)	Regulatory Status (at cutoff)^‡^	Recruitment Status (at cutoff)	Patients: Enrolled/Treated/Evaluable	Median (or reported) follow-up duration	Sponsor/Developer	Key clinical outcome^†^	Safety summary^†^	Evidence source^§^	References
NCT03745287 (CLIMB-121); long-term follow-up in NCT04208529 (CLIMB-131)	Severe sickle cell disease	Exagamglogene autotemcel (exa-cel; brand: Casgevy)	*BCL11A* erythroid-specific enhancer	*Ex vivo* CRISPR-Cas9; electroporation of Cas9 RNP into autologous CD34^+^ HSPCs; re-infusion after busulfan myeloablative conditioning	Phase I/II/III	United Kingdom MHRA 16 November 2023; US FDA December 2023, with indication expanded on 1 July 2026 to patients aged ≥2 years with SCD with recurrent VOCs; EU EMA conditional marketing authorization February 2024; also approved in Saudi Arabia, Switzerland, and Canada	Completed (long-term follow-up ongoing)	Enrolled: 63; treated with exa-cel: 44; primary-efficacy population: 30	Median follow-up: 19.3 months (range, 0.8–48.1) among the 44 treated patients	Vertex Pharmaceuticals; CRISPR Therapeutics	29/30 (97%) of patients in the primary-efficacy population were free from severe vaso-occlusive crises for ≥12 consecutive months, and 30/30 (100%) were free from inpatient hospitalization for severe vaso-occlusive crises for ≥12 consecutive months	All 44 patients experienced at least one adverse event, most of which were grade 1–2; 42/44 (95%) experienced at least one grade 3–4 AE. The safety profile was generally consistent with myeloablative busulfan conditioning and autologous HSPC transplantation. Twenty patients (45%) experienced serious adverse events, none of which were considered related to exa-cel. One death due to COVID-19-related respiratory failure was considered unrelated to exa-cel; no malignancies were reported.	ClinicalTrials.gov; FDA approval documents; EMA EPAR; MHRA assessment report; primary clinical publication	(Frangoul et al., 2021 ; 2024; Kerwash et al., 2024)
NCT03655678 (CLIMB-111); long-term follow-up in NCT04208529 (CLIMB-131)	Transfusion-dependent β-thalassemia	Exagamglogene autotemcel (exa-cel; brand: Casgevy)	*BCL11A* erythroid-specific enhancer	*Ex vivo* CRISPR-Cas9; electroporation of Cas9 RNP into autologous CD34^+^ HSPCs; re-infusion after busulfan myeloablative conditioning	Phase I/II/III	Approved: United Kingdom MHRA 16 November 2023; US FDA January 2024, with indication expanded on 1 July 2026 to patients aged ≥2 years with TDT; EU EMA conditional marketing authorization February 2024; also approved in Saudi Arabia, Switzerland, and Canada	Completed (long-term follow-up ongoing)	Enrolled (actual): 59; Evaluable/treated: 49/52 (94.2%)	Median follow-up: 34.7 months (range 4.5–63.8); transfusion independence maintained up to 5 years (mean 32.4 months; range 14.3–60.8) in TI12 responders	Vertex Pharmaceuticals; CRISPR Therapeutics	49/52 (94.2%) of evaluable patients achieved transfusion independence for ≥12 consecutive months; mean total Hb ≥ 12 g/dL from Month 5 onward; HbF ≥11 g/dL from Month 5 onward with pancellular distribution (≥95% F-cells); 26/56 (46.4%) discontinued iron chelation	Most common AEs: febrile neutropenia (60.7%), headache (55.4%), stomatitis (53.6%); 2 SAEs related to exa-cel (resolved); no deaths, no study discontinuations, no malignancies reported	ClinicalTrials.gov NCT03655678; MHRA Public Assessment Report; FDA BLA 125745 approval letter; EMA EPAR for exagamglogene autotemcel	(Locatelli et al., 2024; de la Fuente et al., 2025)
NCT04601051; NCT06128629; NCT06672237	Hereditary transthyretin amyloidosis with polyneuropathy (ATTRv-PN) and transthyretin amyloidosis with cardiomyopathy (ATTR-CM)	Nexiguran ziclumeran (nex-z; formerly NTLA-2001)	*TTR*	*In vivo* CRISPR-Cas9; LNP delivery of Cas9 mRNA + sgRNA targeting hepatocyte *TTR*	Phase I (NCT04601051; completed); Phase III (NCT06128629, MAGNITUDE, ATTR-CM; NCT06672237, MAGNITUDE-2, ATTRv-PN)	Investigational in all jurisdictions; no marketing authorization issued at cutoff	NCT04601051: completed; NCT06128629 and NCT06672237: recruiting	Registry enrollment: 72 across ATTRv-PN and ATTR-CM cohorts. Initial 2021 report: 6 participants. 2025 ATTRv-PN primary report: 36 treated; mean follow-up 27 months	Initial clinical report: safety assessment through Day 28; subsequent follow-up demonstrated sustained TTR reduction and reported exploratory clinical outcomes	Intellia Therapeutics; Regeneron Pharmaceuticals (collaboration)	Dose-dependent serum TTR reductions of 52% and 87% at Day 28 were observed at 0.1 and 0.3 mg/kg, respectively, with sustained TTR reduction reported during subsequent follow-up; exploratory clinical improvement	Most AEs mild (grade 1–2); transient infusion-related reactions; pre-medication with corticosteroids and antihistamines used to mitigate inflammatory responses	ClinicalTrials.gov; primary clinical publication; Intellia regulatory/development disclosures	(Gillmore et al., 2021 ; 2025; Fontana et al., 2024)
NCT03872479	Leber congenital amaurosis type 10 (LCA10)	EDIT-101	*CEP290* (IVS26 deep-intronic splice variant)	*In vivo* CRISPR-Cas9; subretinal injection of AAV5 vector encoding SaCas9 + CEP290-specific gRNAs in photoreceptor cells	Phase I/II	Investigational in all jurisdictions; no marketing authorization issued at cutoff	Unknown; last known status: active, not recruiting (last registry update: December 2022)	Enrolled (actual): 34; Outcomes reported across dose cohorts (Pierce et al.), n varies by cohort (up to 18 in some analyses)	Median follow-up reported across cohorts varies; 1-year safety and efficacy readouts published in Pierce et al.	Editas Medicine	21% of the patients met the predefined threshold for clinically meaningful improvement in best-corrected visual acuity; no clear dose-response across cohorts; editing efficiency at the molecular level not reported in the published trial	No dose-limiting toxicities; ocular adverse events included subretinal procedure-related findings, including inflammation and retinal changes; no systemic CRISPR-related serious adverse events were reported	ClinicalTrials.gov; primary clinical publication; regulatory/development disclosures	(Maeder et al., 2019; Pierce et al., 2024)
NCT02793856	Advanced/refractory metastatic non-small-cell lung cancer	Autologous PD-1-knockout T-cell therapy (*ex vivo* CRISPR-Cas9 edited autologous T cells)	*PDCD1*	*Ex vivo* CRISPR-Cas9; electroporation of Cas9 + sgRNA targeting exon 2 of *PDCD1* into autologous T cells, followed by expansion and re-infusion	Phase I	Investigational in all jurisdictions; no marketing authorization issued at cutoff	Completed	Enrolled (actual): 12; Response-evaluable: 7	Median progression-free survival: 7.7 weeks (95% CI, 6.9–8.5); median overall survival: 42.6 weeks (95% CI, 10.3–74.9)	Sichuan University	Of 7 response-evaluable patients, 2 of 4 in the 2 × 10^7^/kg cohort achieved stable disease; 3 patients at lower doses progressed; 8-week PFS was 28.6%; novel T-cell clonotypes detected post-infusion, but no objective responses (CR/PR) reported	Treatment-related adverse events were predominantly grade 1–2; no grade ≥3 treatment-related adverse events or cytokine-release syndrome were reported	ClinicalTrials.gov; primary Nature Medicine publication	(Lu et al., 2020)
NCT05120830; NCT06634420 (HAELO)	Hereditary angioedema with C1 inhibitor deficiency	Lonvoguran ziclumeran (lonvo-z; formerly NTLA-2002)	*KLKB1*	*In vivo* CRISPR-Cas9; LNP delivery of Cas9 mRNA + sgRNA targeting hepatocyte *KLKB1*	Phase I/II (NCT05120830); Phase III (NCT06634420, HAELO	Investigational in all jurisdictions; no marketing authorization issued at cutoff	Active, not recruiting	Enrolled (actual): 37 for NCT05120830; Phase III HAELO: 80 randomized; 52 received lonvo-z and 28 received placebo	Median 7.5 months (range, 4.9–12.8) as of 10 February 2026	Intellia Therapeutics	The least-squares mean monthly HAE attack rate from weeks 5–28 was 0.26 with lonvo-z versus 2.10 with placebo, corresponding to an 87% relative reduction (P < 0.001)	AE occurred in 92% of lonvo-z recipients and 86% of placebo recipients; no serious or grade ≥3 adverse events were reported in the lonvo-z group during the primary observation period	ClinicalTrials.gov; Phase III primary clinical publication	(Musunuru et al., 2021; Cohn et al., 2025 ; 2026)

* Information for this table was retrieved from the following primary sources: ClinicalTrials.gov (https://clinicaltrials.gov), the WHO international clinical trials registry platform, the European Union Drug Regulating Authorities Clinical Trials Database, the U.S. food and drug administration approval letters and briefing documents, the European Medicines Agency European Public Assessment Reports, the United Kingdom medicines and healthcare products regulatory agency public assessment reports, peer-reviewed primary publications of each pivotal trial, and peer-reviewed regulatory analyses. Information cutoff date: 31 July 2026. Trial phase and recruitment status reflect the most recent publicly available registry record retrieved before or on the information cutoff date. Where a registry record was not recently updated, the status is reported as ‘unknown’ or as the last known status rather than being interpreted as current.

‡ Regulatory status reflects marketing approvals, conditional approvals, orphan designations, and investigational status active as of the information cutoff date. Subsequent label expansions, modifications, or post-marketing requirements are not captured.

† Key clinical outcomes and safety summaries reflect the most recent peer-reviewed publication or regulatory assessment available at the cutoff date; numerical values are reproduced from the cited primary sources.

§ Evidence Source indicates the primary document type underlying the row (peer-reviewed publication, regulatory assessment report, or both); full source identity is given in the References column. Where multiple records described the same trial, the most recent peer-reviewed publication or regulatory assessment was treated as primary; earlier records were retained only for context.

## 6 Safety, ethical, and regulatory considerations

### 6.1 On-target and off-target genome editing events

In the development of CRISPR-Cas9 therapeutics, a major safety concern is the potential for unintended on- and off-target genome editing events (Lopes and Prasad, 2024). While on-target activity occurs precisely at the intended genomic locus, the resulting repair outcomes are heterogeneous. They include not only the desired therapeutic edit but also unintended repair products, such as large deletions, chromosomal inversions, translocations, or loss of heterozygosity. This unpredictability arises because DSB repair via non-homologous end joining (NHEJ) is inherently error-prone and generates a diverse spectrum of insertions and deletions at the cleavage site. An off-target effect occurs when the Cas9-sgRNA complex binds to and cleaves genomic sequences with partial homology to the intended target site (Doench et al., 2016). Because off-target modifications can create mutations in genes with critical cellular functions (e.g., oncogenes or tumor suppressor genes), the off-target events represent a significant safety risk. Several factors influence the frequency and distribution of off-target activity, including sgRNA design, genome-wide sequence similarity, chromatin accessibility, and the type of Cas nuclease used (Kalter et al., 2025). To reduce these risks, researchers have recently improved their ability to optimize CRISPR specificity. One improvement is the implementation of algorithms that assist in gRNA design by selecting target sequences with minimal homology to other regions of the genome (Doench et al., 2016). Additionally, researchers have made progress in developing engineered, high-fidelity variants of the Cas9 protein (e.g., eSpCas9 and SpCas9-HF1) that can reduce non-specific DNA interactions while retaining highly effective on-target activity.

Researchers have also developed many experimental techniques to detect off-target editing. Examples of these genome-wide profiling techniques include GUIDE-seq, DISCOVER-seq, and CIRCLE-seq. GUIDE-seq and DISCOVER-Seq are cell-based assays with different biological requirements, whereas CIRCLE-seq is an *in vitro* genomic DNA assay; candidate sites require orthogonal validation in the clinically relevant cell type (Tsai et al., 2015; 2017; Wienert et al., 2019). These profiling methods are being incorporated into preclinical safety assessments and regulatory submissions for genome editing therapies. Minimizing off-target activity while achieving efficient therapeutic editing is essential to the safe and effective clinical use of CRISPR-Cas9 technologies. Although these technologies substantially improve off-target detection, they remain limited by sensitivity and sampling and therefore cannot rule out extremely rare editing events.

Importantly, genome editing safety should not be evaluated solely on the basis of conventional off-target mutations. Increasing evidence indicates that even correctly targeted DSBs may produce unintended on-target genomic alterations, including large deletions, chromosomal inversions, translocations, loss of heterozygosity, and complex genomic rearrangements (Lopes and Prasad, 2024). These events arise from error-prone DNA repair pathways following CRISPR-induced cleavage rather than incorrect gRNA targeting. Their measured frequency is highly locus-, cell-, editor-, culture-, and assay-dependent and may be underestimated by short-read or amplicon-based methods. Given their potential biological consequences, including disruption of neighboring genes or regulatory elements and possible oncogenic transformation, comprehensive profiling of both sequence-level and structural genomic integrity is essential during therapeutic development.

### 6.2 Genomic instability and genotoxicity

Genomic instability is a fundamental hallmark of cancer and refers to an increased tendency of the genome to accumulate genetic alterations such as large deletions and chromosomal rearrangements. These genomic changes disrupt normal gene function and activate oncogenes or inactivate tumor suppressor genes, which promote tumor initiation and progression (Martin et al., 2010). While some cancers arise from inherited defects in DNA repair pathways, many develop in cells that initially possess functional repair mechanisms but gradually accumulate DNA damage due to replication stress, environmental exposures, or even aging (Yoshioka et al., 2021). A major contributor to genomic instability is the accumulation of DSBs, which are among the most lethal forms of DNA damage. Cells rely on the DNA damage response network to detect and repair these lesions through pathways such as homologous recombination and non-homologous end joining (Gujar et al., 2025). Defects in these pathways can lead to persistent DNA damage, chromosomal instability, and tumorigenesis. Senescent and aging cells often accumulate DSBs, leading to chromosomal abnormalities and cytosolic DNA fragments that further destabilize the genome and contribute to the increased incidence of cancer with age.

Several hereditary genomic instability syndromes arise from mutations in genes involved in DNA repair and DDR pathways. Defects in mismatch repair genes such as *MSH2*, *MLH1*, and *PMS2* lead to microsatellite instability, which is a common and important feature of Lynch syndrome and a common event in colorectal cancer (Heinen, 2016). MSI can generate frameshift mutations in genes including *TGFBR2*, *IGF2R*, *CDX2*, and *BAX*, thereby promoting tumor progression. Chromosomal instability disorders such as ataxia-telangiectasia, Nijmegen breakage syndrome (pathogenic variants in *NBN*, encoding nibrin/NBS1), Bloom syndrome (BLM helicase defect), and Fanconi anemia are characterized by impaired DSB repair and increased cancer susceptibility. Pathogenic variants in key HR genes, including *BRCA1* and *BRCA2*, increase the risk of breast, ovarian, and other cancers. Similarly, pathogenic variants in *WRN* cause Werner syndrome, a disorder associated with premature aging and genomic instability. Furthermore, pathogenic variants in nucleotide excision repair (NER) genes cause disorders such as xeroderma pigmentosum (for example, *XPA*–*XPG*) and Cockayne syndrome (principally *ERCC6/CSB* and *ERCC8/CSA*), highlighting the indispensable role of these DNA repair pathways in preventing carcinogenesis (Yoshioka et al., 2021).

Recent studies have further demonstrated that CRISPR-induced DSBs may occasionally trigger complex structural genomic abnormalities beyond conventional insertions and deletions. These include chromosomal translocations, chromothripsis, chromosome arm losses, micronucleus formation, and large-scale genomic rearrangements generated during DNA repair (Kosicki et al., 2018b; 2018a). In addition, vector sequences delivered during genome editing may rarely become incorporated at cleavage sites, particularly when DNA-based delivery systems are used (Rui et al., 2019). Although these events are uncommon, their occurrence reinforces the importance of minimizing prolonged nuclease expression and optimizing genome editing strategies that reduce reliance on DSBs, such as base editing and prime editing.

Another important consideration is activation of the p53-mediated DNA damage response following CRISPR-induced DSBs. Although p53 activation serves as a protective mechanism by promoting DNA repair, cell-cycle arrest, or apoptosis, it may also create selective pressure favoring the survival and expansion of edited cells with impaired p53 function (Haapaniemi et al., 2018; Ihry et al., 2018). Such p53-mediated selection has raised theoretical concerns regarding long-term clonal evolution following therapeutic genome editing, particularly in *ex vivo* edited HSCs. Current evidence indicates that these risks are primarily associated with DSB-mediated editing, whereas base editing and prime editing generally reduce, but do not completely eliminate, these potential safety concerns because they largely avoid double-strand DNA cleavage (Dorset and Bak, 2023).

Long-term genomic surveillance is also required because clonal expansion may not become detectable until months or years after treatment. Rare edited clones carrying structural genomic alterations or growth advantages may initially remain below the limit of detection but subsequently expand following transplantation. Consequently, serial molecular monitoring using hematological, cytogenetic, and genomic analyses is required to distinguish stable polyclonal engraftment from emerging clonal dominance and to identify delayed adverse events that cannot be detected during short-term clinical follow-up.

Comprehensive safety evaluation therefore requires complementary analytical approaches. Although short-read next-generation sequencing accurately detects single-nucleotide variants and small insertions or deletions, it has limited sensitivity for identifying large deletions, balanced translocations, inversions, chromothripsis, and other complex structural rearrangements. Product characterization should combine targeted deep sequencing for intended and candidate off-target loci; translocation-aware assays such as CAST-Seq or UDiTaS; long-read sequencing or optical genome mapping for larger structural variants; and cytogenetic or clonal analyses where appropriate. No single assay excludes all rare events (Giannoukos et al., 2018; Kalter et al., 2025).

### 6.3 Immunogenicity and host responses

The clinical application of CRISPR-Cas genome editing has raised important concerns about immunogenicity and host immune responses, which may arise against the Cas9 protein, the delivery vector (particularly viral vectors), or edited cells. Cas9 nucleases are typically derived from bacterial species such as *S. pyogenes or Staphylococcus aureus,* making them a foreign antigen to the human immune system. Cas9 expression may trigger both cell-mediated and humoral immune responses. Pre-existing immunity to these bacterial proteins has been detected in humans, suggesting that immune recognition could limit the efficacy and durability of genome editing therapies. Immune clearance is most concerning when bacterial nuclease or vector antigens are expressed persistently *in vivo*; cells edited *ex vivo* with transient RNP exposure may contain little or no persistent Cas9 antigen after manufacturing (Charlesworth et al., 2019; Wagner et al., 2019). Experimental studies using AAV-mediated Cas9 delivery have been conducted in the central nervous system, primarily focusing on the immunogenic potential of CRISPR components. In adult mice, intracranial AAV-Cas9 administration induced a robust neuroinflammatory response, including microglial and astrocytic activation, increased MHC-II expression, and infiltration by CD3^+^ T cells. These immune responses correlated with reduced neuronal density and loss of detectable Cas9 expression, suggesting that immune-mediated clearance of Cas9-expressing cells occurs (Chew et al., 2016). Administration during early postnatal development results in transient microglial activation, sustained Cas9 expression, and minimal neuronal loss, thereby allowing the immature system to exhibit greater tolerance to CRISPR components. Both neonatal and adult treatments generate Cas9-reactive antibodies that elicit a humoral immune response.

Beyond Cas9 itself, viral delivery vectors, such as AAV, can also stimulate immune reactions through recognition of viral capsid proteins. AAV vectors can provoke clinically important capsid-specific humoral and cellular responses; systemic high-dose exposure can also trigger inflammatory, hepatic, hematologic, or complement-mediated toxicities. Protocol-specific immunosuppression, most commonly corticosteroid-based regimens in selected AAV applications, may be used to mitigate clinically relevant immune responses (Anliker et al., 2022). Early CRISPR clinical trials have reported that most immune-related events were manageable, including transient inflammatory responses, cytokine release, and immune activation associated with vector delivery or edited cells. While severe adverse effects remain rare, these observations highlight the importance of careful monitoring of immune responses in genome editing therapies. Strategies such as engineering low-immunogenic Cas9 variants, transient editor expression, immune modulation, and improved delivery systems are being investigated to minimize host immune responses. Because immune-mediated clearance of edited cells may affect long-term therapeutic durability, post-treatment monitoring should assess not only immunogenicity but also the persistence of edited cell populations, sustained therapeutic benefit, and the potential emergence of delayed adverse events. Such longitudinal surveillance will become increasingly important as more patients receive genome editing therapies in routine clinical practice.

### 6.4 Ethical, social, and equity issues

Rapid advances in CRISPR-Cas9 genome editing technologies have raised significant ethical and social concerns, particularly regarding their application in human germline editing and reproductive medicine. Heritable human genome editing (HHGE) refers to the modification of gametes, their precursors, or embryos with the intention or possibility that the edited nuclear DNA will be transmitted to future generations. Crucially, *in vitro* laboratory germline research that is not intended to establish a pregnancy must be strictly distinguished from clinical heritable editing. Although heritable genome editing has been proposed as a theoretical means of preventing transmission of severe genetic disorders, its clinical use remains ethically and scientifically unacceptable or prohibited/restricted in many jurisdictions. Concerns include the risk of unintended mutations, an incomplete understanding of gene-environment interactions, and the possibility of facilitating genetic enhancement of non-disease traits, such as intelligence or physical characteristics (Baylis et al., 2020; Doudna, 2020). Beyond germline modification, ethical considerations extend to embryo research and the reproductive applications of CRISPR, including the moral status of the embryo and the acceptable boundaries of scientific experimentation.

Another major concern involves equitable access to CRISPR-based therapies. Although genome editing offers promising treatments for genetic disorders, the high cost of research and development, individualized GMP manufacturing, myeloablative conditioning, hospitalization, specialized infrastructure, and long-term follow-up may limit access to wealthy healthcare systems and patients. For example, early CRISPR-based therapies have been approved for conditions such as SCD, which have extremely high treatment costs, raising concerns regarding affordability, healthcare disparities, and equitable access, particularly in low- and middle-income countries, without appropriate policy frameworks and international collaboration (Frangoul et al., 2021). Ensuring global health equity will also require strategies such as international funding initiatives, technology sharing, and policy frameworks that promote the fair distribution of genome editing technologies. Addressing these ethical and social challenges through inclusive dialogue or healthcare policies will be essential to ensuring that the benefits of CRISPR technologies are realized in a safe, just, and globally accessible manner (Baylis et al., 2020). Table 4 presents the major safety risks and mitigation strategies for CRISPR-Cas9 technologies.

**TABLE 4:** Major safety risks and mitigation strategies.

Risk category	Mechanism/Examples	Detection Methods	Mitigation Strategies	Clinical evidence	References
Off-target effects	Cas9 binds similar sequences → unintended mutations	GUIDE-seq, DISCOVER-seq, CIRCLE-seq	High-fidelity Cas9, optimized sgRNA	High-fidelity Cas9 variants have demonstrated substantially reduced off-target cleavage in experimental studies while retaining useful on-target activity	(Doench et al., 2016; Tsai et al., 2017; Guo et al., 2023)
On-target errors	Indels, large deletions at target site	Deep sequencing, genome profiling	Use of DSB-independent editors where mechanistically appropriate, transient editor exposure, optimized nuclease design, and target-specific structural-variant assessment	Base and prime editing reduce large deletions and genomic rearrangements associated with double-strand breaks	(Kosicki et al., 2018b; Aussel et al., 2025)
Genomic instability	Double-strand DNA breaks may induce large deletions, chromosomal rearrangements, translocations, and chromothripsis	Karyotyping, whole-genome sequencing	Avoid DSB (base/prime editing)	Rare genomic rearrangements have been reported, highlighting the need for long-term genomic surveillance	(Charlesworth et al., 2019; Wagner et al., 2019)
Immunogenicity	Immune response to Cas9 or viral vectors	Cytokine assays, antibody detection	Immunosuppression, transient expression	Early clinical trials have generally reported manageable immune responses, although pre-existing immunity to Cas proteins remains a potential concern	(Chew et al., 2016; Gujar et al., 2025)
Delivery-related toxicity	Immune activation, hepatotoxicity, complement activation, and nanoparticle-associated toxicity	Clinical monitoring, biodistribution studies	Dose optimization, transient delivery, biodegradable nanoparticles, and improved vector engineering	Early clinical studies of LNP-mediated *in vivo* editing have demonstrated generally manageable short-term safety profiles, although infusion reactions and other formulation- or dose-dependent toxicities require continued monitoring	(Gillmore et al., 2021; Fontana et al., 2024)
Ethical concerns	Current international guidelines restrict clinical germline genome editing while permitting tightly regulated somatic gene editing applications	Regulatory review, ethical boards	Policy frameworks, restricted use	Germline editing widely restricted	(Baylis et al., 2020; Doudna, 2020)
Long-term safety	Delayed adverse events, clonal expansion, secondary malignancies, or unforeseen genomic alterations	Long-term follow-up (10–15 years)	Long-term clinical follow-up, genomic surveillance, and pharmacovigilance	Regulatory agencies recommend long-term follow-up (up to 15 years) for genome editing therapies	(Anliker et al., 2022)

### 6.5 Regulatory landscape of CRISPR-based therapeutics

The rapid translation of CRISPR-Cas genome editing technologies into clinical applications has spurred the development of comprehensive regulatory frameworks to ensure the safety, efficacy, quality, and manufacturing consistency of gene-edited therapeutic products. Early clinical evidence supporting therapeutic efficacy has particularly helped in treating inherited blood disorders such as SCD and β-thalassemia, which have set important regulatory precedents for CRISPR-based medicine (Frangoul et al., 2021). These initial approvals demonstrated the feasibility of *ex vivo* genome editing approaches targeting HSCs, providing a foundation for regulatory agencies to evaluate similar therapies. In the United States and European Union, CRISPR-based therapeutics are regulated within established frameworks for human gene therapy and advanced therapy medicinal products (ATMPs), respectively. Regulatory assessment encompasses product characterization, manufacturing and quality control, nonclinical safety, genomic integrity, clinical efficacy, and long-term risk monitoring (Anliker et al., 2022).

The regulatory landscape continues to evolve as CRISPR applications have expanded across diverse disease areas, including cancer, metabolic disorders, and infectious diseases. Clinical programs include *in vivo* editing for inherited or metabolic diseases such as ATTR amyloidosis and *ex vivo* immune-cell editing for hematologic and solid malignancies. These advancements present unique regulatory challenges, particularly in assessing long-term safety and potential off-target effects, including the risk of unintended genomic alterations. Regulatory agencies emphasize comprehensive nonclinical evaluation, including genome-wide off-target analysis, validation studies, and assessment of genomic stability before clinical approval. Evolving regulatory guidance also reflects differences in the therapeutic context. For inherited genetic disorders, the principal objective is durable disease modification or long-term therapeutic benefit. Regulators may accept smaller, single-arm trials because of the limited patient population. Trial design is indication- and phase-specific; while many early oncology cell-therapy studies utilize single-arm designs, comparative trials are generally required to establish definitive efficacy for broader clinical use. Because of the permanent nature of genomic modifications, regulatory agencies such as the U.S. FDA recommend risk-based long-term follow-up (LTFU) extending up to 15 years. The precise duration of this surveillance is dictated by the therapeutic product’s persistence, integration potential, *in vivo* biodistribution, targeted cell lifespan, and anticipated genotoxic risks (Anliker et al., 2022; Eisenman and Swindle, 2022).

## 7 Technological advances shaping future therapeutics

### 7.1 Next-generation CRISPR systems

Next-generation CRISPR technologies have expanded genome editing beyond the conventional Cas9 system, enabling more precise and versatile manipulation of genetic material. Novel CRISPR-associated nucleases, such as Cas12 and Cas13, have expanded the genome editing repertoire by introducing distinct mechanisms of action. Cas12a is a class 2 type V nuclease that recognizes T-rich PAMs and usually generates staggered DSBs with 5′overhangs, thereby enabling access to target sites not compatible with the canonical SpCas9 NGG PAM (Pickar-Oliver and Gersbach, 2019). In contrast, Cas13 targets RNA rather than DNA, enabling transcriptome engineering and transient gene regulation without permanent genomic alteration. These RNA-targeting systems are particularly valuable for studying gene expression and dynamics, as well as for developing antiviral therapeutics. Ongoing discovery of smaller Cas variants, such as *S. aureus* Cas9 (SaCas9) and CasX (now commonly classified as Cas12e), has addressed delivery limitations, particularly for packaging-constrained vectors like AAV, improving the feasibility of *in vivo* genome editing applications.

Recent developments have also aimed to reduce reliance on DSB formation because intended DSBs can generate large deletions and structural rearrangements, while nuclease targeting at unintended loci creates a separate off-target genotoxicity risk. Base editors consist of catalytically impaired Cas proteins fused to a deaminase and enable targeted single-nucleotide conversions without inducing obligate DSBs, thereby reducing selected DSB-associated genotoxicity risks while retaining editor-specific liabilities such as bystander and off-target deamination. Prime editing combines a Cas9 nickase with a reverse transcriptase and a prime-editing gRNA (pegRNA), enabling all 12 possible single-base substitutions in principle, together with targeted small insertions and deletions, without requiring an exogenous donor DNA template or an intentional DSB. Thus, base and prime editing can reduce selected DSB-associated genomic liabilities, although both platforms retain distinct unintended-editing and delivery-related risks that require product-specific evaluation (Komor et al., 2016; Gaudelli et al., 2017; Anzalone et al., 2019). Advances in the CRISPR system have enabled engineering approaches, including multiplexed editing platforms and dual-guide systems, that enable large-scale genetic interaction studies and combinatorial gene targeting, particularly in cancer research. Compared with conventional CRISPR-Cas9, next-generation genome editing technologies offer greater editing precision while minimizing reliance on double-strand break formation, thereby reducing the risk of unintended genomic alterations. As a result, base editing and prime editing are increasingly considered for therapeutic applications that require precise correction of pathogenic variants, whereas conventional CRISPR-Cas9 remains particularly effective for gene disruption strategies.

### 7.2 Improved delivery platforms

Efficient and targeted delivery remains a major challenge in the clinical translation of CRISPR-based therapeutics. Recent advances have focused primarily on developing delivery systems that enhance targeting specificity, improve stability, and facilitate intracellular delivery. Among all of these, LNPs have emerged as one of the most extensively investigated non-viral platforms. LNPs are designed with ionizable lipids and stimuli-responsive features that enhance endosomal escape and intracellular release of CRISPR components. Surface modification using cell-targeting ligands, such as peptides, antibodies, or receptor-binding molecules, enables selective delivery to target tissues and increases editing efficiency while reducing off-target effects (Rostami et al., 2024). In addition to LNPs, hybrid delivery systems combining viral and nonviral characteristics are gaining attention. Virus-like particles and engineered extracellular vesicles are being investigated as transient delivery platforms that may reduce some limitations of conventional viral-vector delivery, although their biodistribution, immunogenicity, manufacturing reproducibility, and clinical performance remain under evaluation. Other nanocarriers, including polymeric nanoparticles, gold nanoparticles, and DNA nanostructures, exhibit tunable physicochemical properties that can be engineered for controlled release, improved stability, and tissue-specific targeting.

A critical focus of next-generation delivery strategies is overcoming biological barriers, such as the blood-brain barrier and the tumor microenvironment. The blood-brain barrier restricts the entry of large biomolecules by posing a significant challenge for treating neurological diseases. Upcoming approaches, including receptor-mediated transport, ligand-functionalized nanoparticles, and exosome-based systems, are being developed to facilitate intracellular delivery of CRISPR components across the blood-brain barrier (Alsaiari et al., 2024). Solid tumors, with barriers such as dense extracellular matrices and abnormal vasculature, usually limit nanoparticle penetration. Strategies such as nanoparticle size optimization, tumor-penetrating peptides, and microenvironment-responsive carriers are being explored more extensively to improve tumor targeting and distribution.

### 7.3 Functional genomics and synthetic lethality screens

Synthetic lethality arises when the simultaneous loss of function of two genes causes cell death, whereas loss of either gene alone is compatible with cell survival. CRISPR-Cas9-based functional genomic screening enables systematic, genome-wide identification of such synthetic lethal gene pairs, providing a scalable approach for discovering context-specific therapeutic vulnerabilities in cancer (Wu et al., 2025). For example, genome-wide CRISPR screening identified *KAT2A* as selectively essential for the survival of acute myeloid leukemia cells; genetic inactivation of *KAT2A* induced leukemic cell death and promoted differentiation toward a more normal phenotype (Tzelepis et al., 2016). Integrating CRISPR screening data with large-scale genomic resources such as The Cancer Genome Atlas further facilitates prioritization of clinically relevant targets and improves understanding of polygenic cooperativity within tumors, particularly for identifying therapeutic vulnerabilities in treatment-refractory cancers. CRISPR-based functional screening has similarly been applied to inherited disorders, for example, to identify genetic modifiers that can reactivate HbF expression as a therapeutic strategy for hemoglobinopathies. In cancer models lacking the tumor suppressor SMAD4, genome-wide CRISPR screening identified genes selectively required for the survival of SMAD4-deficient cells; disruption of these genes induced selective cell death, providing candidate targets for genotype-selective cancer therapy (Feng et al., 2022). More broadly, genome-wide CRISPR screens have identified genetic determinants of drug sensitivity, informing strategies to enhance the efficacy of existing therapeutic agents and to identify combination targets in treatment-resistant disease. Collectively, these functional genomic approaches are advancing the identification of context-specific therapeutic vulnerabilities and supporting the development of mechanism-based, individualized treatment strategies for cancer and inherited genetic disorders.

### 7.4 Personalized and multiplex genome editing

CRISPR-Cas9 enables the design of gRNAs directed toward disease-associated genomic variants, thereby supporting mutation-specific genome editing and individualized therapeutic strategies. Depending on the molecular defect and the selected editing platform, targeted genomic modification can be used to disrupt pathogenic alleles, modify regulatory elements, or restore gene function. In hemoglobinopathies, for example, CRISPR-based disruption of the erythroid-specific *BCL11A* enhancer promotes HbF expression and provides a disease-modifying therapeutic mechanism without directly correcting the underlying *HBB* mutation (Psatha et al., 2018; Frangoul et al., 2024). Newer DSB-independent platforms, including base and prime editing, extend this targeted approach while avoiding double-strand breaks, thereby reducing the risk of chromosomal rearrangements associated with conventional nuclease-based editing (Li et al., 2023).

Multiplex genome editing further permits simultaneous modification of multiple genomic loci within the same therapeutic cell population. This capability is particularly relevant to engineered immune-cell therapies, where simultaneous disruption of inhibitory pathways, endogenous T cell receptor loci, and other genes can be combined with insertion of tumor-targeting receptors. Such multiplex strategies may improve the functional properties and persistence of engineered immune cells, although their clinical application requires rigorous assessment of editing efficiency, genomic integrity, and product consistency (Sharma and Giri, 2024). Emerging technologies such as PASTE, which couples prime-editing-derived targeting with integrase activity to support programmable large-cargo insertion without classical HDR, further expand the scope of personalized genome correction to conditions caused by large deletions or complex structural variants (Yarnall et al., 2023). The approach remains preclinical and faces constraints in delivery, efficiency, insertion fidelity, and off-target integration. Similarly, in DMD, sgRNAs designed against common mutational hotspots are being investigated to restore the dystrophin reading frame in a mutation-specific manner. However, many of these technologies remain at the preclinical or early translational stage. Accordingly, individualized genome editing should be regarded as a developing precision-medicine strategy whose clinical applicability depends on disease biology, target site characteristics, delivery feasibility, editing specificity, and long-term safety rather than as a universally applicable therapeutic approach.

## 8 Integration of CRISPR-Cas9 into clinical practice

### 8.1 Patient selection and stratification

Successful clinical implementation of CRISPR-Cas9 therapeutics requires accurate molecular diagnosis and comprehensive genomic characterization to identify patients whose disease biology, target genotype, and clinical characteristics match the intended editing strategy. Patient selection generally relies on validated molecular diagnostics, sequencing, disease-specific biomarkers, and confirmation that the pathogenic variant and target sequence are compatible with the therapeutic construct. Biomarker-based stratification and functional genomic screening may further support prediction of therapeutic response and identification of molecular subgroups that are most likely to benefit from a particular editing strategy (Kim and Lee, 2023).

In oncology, monitoring measurable residual disease (MRD) and tumor evolution is essential to assess treatment efficacy and inform clinical decisions. Advanced liquid biopsy platforms, such as SPEAR, enable the real-time detection of cancer-associated single nucleotide variants (SNVs) and dynamic clonal evolution (Bai et al., 2025). Concurrently, novel delivery frameworks, such as Selective Organ Targeting (SORT) nanoparticles, are expanding therapeutic applicability by enabling precise routing of CRISPR components to specific extrahepatic tissues, such as the lungs or spleen (Cheng et al., 2020). Alongside these advances, rigorous patient selection must account for interindividual genetic variation, as patient-specific sequence differences can critically influence gRNA recognition, on-target cleavage efficiency, and the spectrum of potential off-target sites. Thorough genomic characterization ensures the administration of CRISPR-based treatments, maximizing therapeutic outcomes while minimizing safety risks. Integration of molecular diagnostics, genomic profiling, and validated predictive biomarkers may improve patient selection and treatment monitoring for genome editing therapies, although further clinical validation remains necessary. Continued advances in genome editing technologies, delivery systems, and clinical evidence are expected to further expand the therapeutic potential of precision medicine (Cancellieri et al., 2023). As summarized in Table 1, selecting an appropriate genome editing and gene-addition platform should be individualized based on disease biology, target tissue accessibility, delivery feasibility, safety profile, and current clinical maturity. These considerations are central to patient selection and clinical implementation of CRISPR-based therapeutics.

### 8.2 Manufacturing, logistics, and scalability

Autologous genome-edited cell therapies require individualized manufacturing workflows that include cell collection, product-specific enrichment or activation, genome editing, any required culture/expansion, product characterization, release testing, cryopreservation or transport, conditioning where applicable, and reinfusion (Abou-el-Enein et al., 2021). These multistep processes contribute to prolonged manufacturing intervals, substantial infrastructure requirements, and high treatment costs, which may be particularly problematic for patients with rapidly progressive disease. The development of allogeneic or “off-the-shelf” CAR-T products aims to reduce manufacturing time and enable standardized production at scale (Jinka, 2022). CRISPR-based strategies in this setting may involve disrupting endogenous T cell receptor loci to reduce alloreactivity, along with additional genetic modifications intended to improve persistence or reduce immune-mediated rejection. However, allogeneic products introduce distinct challenges, including graft-versus-host disease, host-versus-graft immune responses, genomic safety, and the need to establish reproducible product-release criteria (Abou-el-Enein et al., 2021).

Clinical implementation additionally requires standardized GMP procedures for the preparation, handling, storage, transportation, and administration of genome-edited cell products and CRISPR delivery components. Temperature-controlled storage and transport, specialized manufacturing infrastructure, validated quality control assays, and coordinated supply chain systems are essential for maintaining product identity, potency, sterility, and stability. These requirements may create substantial implementation barriers in healthcare systems with limited cell processing capacity and infrastructure. Non-viral delivery platforms, including LNPs, polymeric carriers, and virus-like particles, may provide opportunities to simplify manufacturing and reduce dependence on integrating viral vectors (Ren et al., 2022); however, tissue-specific delivery, biodistribution, immunogenicity, and reproducible therapeutic exposure remain important translational challenges.

### 8.3 Long-term follow-up and post-treatment surveillance

Because therapeutic genome editing is intended to produce durable or permanent molecular changes, assessment of safety and efficacy must extend beyond the initial treatment period. Delayed adverse events may emerge months or years after treatment and may not be detectable in the relatively small cohorts and limited follow-up periods of early-phase clinical trials. Long-term surveillance should therefore evaluate the durability of therapeutic benefit together with potential delayed genotoxicity, immune-mediated complications, clonal abnormalities, and other treatment-specific adverse events. The duration and intensity of follow-up should be determined according to the editing modality, target cell or tissue, persistence of the therapeutic product, delivery platform, and anticipated biological risks rather than applying a uniform monitoring period to all genome editing therapies (Anliker et al., 2022; Youssef et al., 2026).

Monitoring requirements differ substantially between *ex vivo* and *in vivo* genome editing approaches. For *ex vivo* therapies involving hematopoietic stem and progenitor cells or immune cells, longitudinal assessment should include persistence and function of the edited cell population, hematologic recovery, clonal composition, and surveillance for delayed cytopenias, clonal dominance, or secondary malignancies. Genomic monitoring may additionally be required when there is a plausible risk of persistent or expanding clones carrying unintended sequence or structural alterations (Ferrari et al., 2023; Youssef et al., 2026). In contrast, follow-up after *in vivo* genome editing should focus on the durability of the intended molecular and clinical effect, target-organ function, immune responses to the editor or delivery vehicle, and potential delayed toxicity in both target and non-target tissues. These requirements are particularly important for delivery platforms that result in prolonged exposure to genome editing components or cannot be readily retracted after administration (Anliker et al., 2022).

Effective post-treatment surveillance will therefore require coordinated pharmacovigilance, long-term observational studies, and patient registries capable of integrating clinical outcomes with molecular and genomic safety data. Standardized approaches for detecting clonal evolution, structural genomic abnormalities, immune responses, and loss of therapeutic efficacy would improve comparison of safety outcomes across products and clinical studies. International harmonization of long-term monitoring frameworks may additionally facilitate detection of rare adverse events that would be difficult to identify within individual trials or treatment centers. As CRISPR-based therapeutics enter broader clinical use, sustained follow-up will be essential not only to identify delayed safety signals but also to establish the durability of therapeutic benefit and refine the long-term benefit-risk profile of individual genome editing products (Anliker et al., 2022; Youssef et al., 2026).

### 8.4 Clinical implementation, health economics, and equitable access

Successful translation of CRISPR-based therapeutics into routine clinical practice will depend on the ability of healthcare systems to support complex manufacturing, product testing, treatment delivery, and long-term patient management. This challenge is particularly relevant to autologous *ex vivo* therapies, which require individualized workflows involving cell collection, genome editing, expansion or processing, product characterization, release testing, cryopreservation, transportation, conditioning where applicable, and subsequent reinfusion. These multistep processes increase manufacturing time, infrastructure requirements, and operational complexity and may restrict treatment to specialized centers with advanced cell processing and clinical capabilities (Abou-el-Enein et al., 2021; Lee and Chang, 2024). Greater automation, standardized manufacturing platforms, validated release assays, and expansion of decentralized or regional manufacturing capacity may improve scalability while maintaining product quality and regulatory compliance. Allogeneic and other standardized therapeutic platforms may further simplify selected manufacturing workflows, although they introduce distinct challenges related to immunologic compatibility, persistence, genomic safety, and product consistency.

Economic considerations represent an additional barrier to widespread implementation. Cell and gene therapies often involve substantial upfront costs due to complex development, individualized or specialized manufacturing, quality control requirements, and highly specialized clinical delivery. Moreover, assessing their economic value is complicated by uncertainty about the long-term durability of benefit at the time of regulatory approval, particularly when pivotal clinical studies involve small populations or relatively short follow-up periods (Abuloha et al., 2024). Consequently, conventional reimbursement models developed for repeatedly administered pharmaceuticals may not adequately accommodate potentially one-time therapies with high initial costs and benefits expected to accrue over many years. Health technology assessment, outcome-based reimbursement, staged or performance-linked payment models, and continued collection of real-world effectiveness data may therefore become increasingly important for aligning treatment cost with demonstrated long-term clinical value (Beswick, 2024; Han et al., 2026).

Equitable access remains a major translational challenge because the infrastructure required for genome-edited cell therapies and other advanced therapeutic medicinal products is concentrated predominantly in specialized healthcare systems. Geographic limitations, manufacturing capacity, treatment center availability, affordability, reimbursement policies, and requirements for prolonged follow-up may collectively restrict access, particularly in resource-limited settings. Addressing these disparities will require approaches that extend beyond reduction of product price alone, including technology transfer, expansion of regional manufacturing and treatment capacity, workforce development, harmonization of regulatory and quality standards, and establishment of sustainable systems for long-term patient monitoring. The future clinical impact of CRISPR therapeutics will therefore depend not only on improvements in editing efficiency and safety but also on scalable manufacturing, sustainable financing, appropriate healthcare infrastructure, and equitable access to both treatment and long-term follow-up (Laurent et al., 2024; Han et al., 2026).

## 9 Conclusion

CRISPR-Cas9 has rapidly progressed from a genome editing tool used in basic research to a clinically relevant therapeutic platform with significant potential to treat cancer and inherited genetic disorders. Its clinical potential is exemplified by the regulatory approval of exagamglogene autotemcel for SCD and TDT, as well as encouraging outcomes from early-stage clinical trials in oncology and *in vivo* genome editing applications. These advances demonstrate the feasibility of therapeutically targeted somatic genome modification in selected diseases, while also highlighting the need to characterize heterogeneous on-target and off-target outcomes. Despite these achievements, several challenges continue to limit widespread clinical translation. Key concerns include long-term safety, including potential genotoxicity, off-target effects, immune responses to CRISPR components, and the need for efficient, tissue-specific delivery systems. In addition, manufacturing scalability, regulatory complexity, cost, and equitable access remain significant barriers to broader clinical implementation. Ongoing technological innovations are expected to address many of these limitations. Advances in high-fidelity CRISPR nucleases, base and prime editing, improved delivery platforms, and scalable manufacturing strategies are enhancing the precision, safety, and applicability of genome editing therapies. Together with continued rigorous clinical evaluation, these developments may expand clinical applicability if product-specific delivery, efficacy, genomic safety, manufacturing, and long-term surveillance requirements are met.
