# Supplementary material for: Clinical translation of CRISPR-Cas9 therapeutics in cancer and inherited genetic disorders
Source: Front Genome Ed. 2026 Sep 10;8:1868779. doi: 10.3389/fgeed.2026.1868779 (PMC13601907; doi:10.3389/fgeed.2026.1868779)
Supplement: Supplementary file 1 [file Table1.docx]

**Supplementary Material**

**Supplementary Table S1.** Literature search strategy used for this structured narrative review

| **Source/Database** | **Search purpose** | **Exact search string/source-specific query** | **Evidence/search cutoff date** | **Notes** |
| --- | --- | --- | --- | --- |
| PubMed | Broad search for CRISPR-Cas9 therapeutic applications in cancer and inherited genetic disorders | (“CRISPR-Cas Systems”[Title/Abstract] OR “CRISPR-Cas9”[Title/Abstract] OR “CRISPR-Cas”[Title/Abstract] OR CRISPR[Title/Abstract] OR “clustered regularly interspaced short palindromic repeats”[Title/Abstract]) AND (“gene editing”[Title/Abstract] OR “genome editing”[Title/Abstract] OR “genome engineering”[Title/Abstract] OR “gene therapy”[Title/Abstract]) AND (cancer[Title/Abstract] OR oncology[Title/Abstract] OR neoplasm*[Title/Abstract] OR tumor*[Title/Abstract] OR tumour*[Title/Abstract] OR “solid tumor*”[Title/Abstract] OR leukemia[Title/Abstract] OR lymphoma[Title/Abstract] OR “hematologic malignan*”[Title/Abstract] OR “inherited genetic disorder*”[Title/Abstract] OR “genetic disease*”[Title/Abstract] OR “inherited disease*”[Title/Abstract] OR monogenic[Title/Abstract] OR hereditary[Title/Abstract]) | 31 July 2026 | Principal bibliographic entry search |
| PubMed | Clinical and translational CRISPR-Cas9 studies | (“CRISPR-Cas9”[Title/Abstract] OR “CRISPR-Cas”[Title/Abstract]) AND (“clinical trial”[Title/Abstract] OR clinical[Title/Abstract] OR therapeutic[Title/Abstract] OR treatment[Title/Abstract] OR translational[Title/Abstract]) AND (cancer[Title/Abstract] OR oncology[Title/Abstract] OR “genetic disorder*”[Title/Abstract] OR “inherited disease*”[Title/Abstract]) | 31 July 2026 | Targeted supplementation to identify clinical and translational studies |
| PubMed | *In vivo* CRISPR-Cas9 therapeutic applications and delivery platforms | (“CRISPR-Cas9”[Title/Abstract] OR “CRISPR genome editing”[Title/Abstract]) AND (“in vivo”[Title/Abstract] OR delivery[Title/Abstract] OR “lipid nanoparticle*”[Title/Abstract] OR LNP[Title/Abstract] OR AAV[Title/Abstract] OR “adeno-associated virus”[Title/Abstract]) AND (therap*[Title/Abstract] OR treatment[Title/Abstract] OR clinical[Title/Abstract]) | 31 July 2026 | Targeted search for *in vivo* therapeutic applications and clinically relevant delivery approaches |
| PubMed | *Ex vivo* CRISPR-Cas9 cell therapies and cancer immunotherapy | (“CRISPR-Cas9”[Title/Abstract] OR CRISPR[Title/Abstract]) AND (“ex vivo”[Title/Abstract] OR “T cell”[Title/Abstract] OR “T-cell”[Title/Abstract] OR CAR-T[Title/Abstract] OR “CAR T”[Title/Abstract] OR “NK cell*”[Title/Abstract] OR “hematopoietic stem cell*”[Title/Abstract]) AND (cancer[Title/Abstract] OR oncology[Title/Abstract] OR leukemia[Title/Abstract] OR lymphoma[Title/Abstract] OR immunotherap*[Title/Abstract]) | 31 July 2026 | Targeted supplementation for engineered immune-cell and hematopoietic stem cell therapies |
| PubMed | Inherited genetic disorders and clinically advanced genome-editing therapies | (“CRISPR-Cas9”[Title/Abstract] OR “CRISPR-Cas”[Title/Abstract] OR “genome editing”[Title/Abstract]) AND (“sickle cell”[Title/Abstract] OR thalassemia[Title/Abstract] OR “β-thalassemia”[Title/Abstract] OR “beta-thalassemia”[Title/Abstract] OR “transthyretin amyloidosis”[Title/Abstract] OR “hereditary angioedema”[Title/Abstract] OR “Leber congenital amaurosis”[Title/Abstract] OR “genetic disorder*”[Title/Abstract] OR “inherited disease*”[Title/Abstract]) | 31 July 2026 | Targeted search for major inherited genetic disorders represented in clinical CRISPR development |
| PubMed | Genome editing safety, off-target effects, genomic integrity, and long-term risks | (“CRISPR-Cas9”[Title/Abstract] OR “CRISPR genome editing”[Title/Abstract]) AND (“off-target”[Title/Abstract] OR “off target”[Title/Abstract] OR “genomic rearrangement*”[Title/Abstract] OR “genomic stability”[Title/Abstract] OR “genome integrity”[Title/Abstract] OR immunogenicity[Title/Abstract] OR “long-term safety”[Title/Abstract] OR “long-term follow-up”[Title/Abstract]) AND (therapeutic[Title/Abstract] OR clinical[Title/Abstract] OR human[Title/Abstract]) | 31 July 2026 | Targeted safety and translational risk search |
| PubMed | Emerging editing platforms relevant to future therapeutic applications | (“base editing”[Title/Abstract] OR “prime editing”[Title/Abstract] OR “Cas12”[Title/Abstract] OR “Cas13”[Title/Abstract] OR “next-generation CRISPR”[Title/Abstract]) AND (therapeutic[Title/Abstract] OR clinical[Title/Abstract] OR “gene therapy”[Title/Abstract] OR cancer[Title/Abstract] OR “genetic disorder*”[Title/Abstract]) | 31 July 2026 | Targeted supplementation for emerging genome editing platforms discussed in the technology and future perspectives sections |
| Scopus | Broad multidisciplinary literature search | TITLE-ABS-KEY((“CRISPR-Cas9” OR “CRISPR-Cas” OR CRISPR) AND (“gene editing” OR “genome editing” OR “gene therapy”) AND (cancer OR oncology OR neoplasm* OR tumor* OR tumour* OR “genetic disorder*” OR “inherited disease*” OR monogenic OR hereditary)) | 31 July 2026 | Used as a supplementary multidisciplinary bibliographic database to identify relevant studies not retrieved through PubMed/PMC |
| Web of Science Core Collection | Broad multidisciplinary and citation-based literature search | TS=((“CRISPR-Cas9” OR “CRISPR-Cas” OR CRISPR) AND (“gene editing” OR “genome editing” OR “gene therapy”) AND (cancer OR oncology OR neoplasm* OR tumor* OR tumour* OR “genetic disorder*” OR “inherited disease*” OR monogenic OR hereditary)) | 31 July 2026 | Supplementary bibliographic search and identification of relevant records across biomedical and multidisciplinary journals |
| Google Scholar | Supplementary retrieval of relevant publications and recent/online-first literature | “CRISPR-Cas9” AND (“cancer” OR “genetic disorder” OR “inherited disease”) AND (“clinical trial” OR therapeutic OR treatment) | 31 July 2026 | Used as a supplementary source to identify additional relevant publications, particularly recent and online-first records. Screening was restricted to publications relevant to the review scope |
| ClinicalTrials.gov | Identification and verification of completed, ongoing, recruiting, and registered clinical trials | “CRISPR” OR “CRISPR-Cas9” OR “genome editing”; supplemented by condition-specific searches for cancer, sickle cell disease, β-thalassemia, transthyretin amyloidosis, Leber congenital amaurosis, and hereditary angioedema | 31 July 2026 | Used to identify and verify clinical trial identifiers, study phase, recruitment status, enrollment, and other trial characteristics reported in Table 3 |
| United States Food and Drug Administration (U.S. FDA) | Verification of regulatory approvals and regulatory status of CRISPR-based therapeutics | Official FDA searches for Casgevy (exagamglogene autotemcel) and other CRISPR/genome editing therapeutic products; supplemented by relevant biologics license and regulatory guidance documents | 31 July 2026 | Used for verification of U.S. regulatory status, indication, approval date, and relevant regulatory considerations |
| European Medicines Agency (EMA) | Verification of European regulatory status | Official EMA searches for Casgevy (exagamglogene autotemcel) and relevant advanced therapy medicinal product records | 31 July 2026 | Used to verify European marketing authorization/conditional authorization and regulatory information |
| UK Medicines and Healthcare products Regulatory Agency (MHRA) | Verification of UK regulatory status | Official MHRA searches for Casgevy (exagamglogene autotemcel) and relevant regulatory assessment documents | 31 July 2026 | Used to verify UK authorization and regulatory assessment information |

Notes:

Search methodology: The searches were designed as a structured narrative literature search rather than as a formal systematic review. PubMed/PMC served as the principal biomedical bibliographic database, while Scopus, Web of Science, and Google Scholar were used for supplementary literature retrieval. ClinicalTrials.gov was searched separately to identify and verify registered clinical trials. Official regulatory sources, including the U.S. FDA, EMA, and UK MHRA, were consulted to verify regulatory status and approval information.

Evidence cutoff date: Searches and evidence retrieval were restricted to records and information available through 31 July 2026, which was defined a priori as the evidence cutoff date. No lower publication-date limit was applied; therefore, records from database inception through 31 July 2026 were eligible for consideration.

Search adaptation: Search terms were adapted across databases according to database-specific syntax and indexing requirements. Targeted supplementary searches were performed for clinically important indications, therapeutic approaches, delivery platforms, safety considerations, and emerging genome editing technologies.

Clinical and regulatory verification: Trial characteristics, including registry identifiers, study phase, recruitment status, and enrollment, were cross-checked against ClinicalTrials.gov using information available through 31 July 2026. Regulatory claims were verified using official FDA, EMA, and MHRA sources available through the same cutoff date.

Eligibility and screening: Study eligibility, exclusion criteria, screening procedures, and the distinction between primary clinical evidence, preclinical studies, reviews, and regulatory documents are described in Section 2.1 of the manuscript.
